# Highly Robust, Compressible, Anisotropic, and Fire-Retardant Polyimide/Hydroxyapatite Nanowires/Reduced Graphene Oxide Aerogel for Rapid Adsorption of Viscous Oil Assisted by Sunlight

**DOI:** 10.34133/research.0512

**Published:** 2024-10-29

**Authors:** Pan Huang, Yongxiang Sun, Lin Yang, Haoyu Yang, Ying Hu, Jifang Liu, Xuwen Peng, Hongbo Zeng

**Affiliations:** ^1^ Department of Chemical and Materials Engineering, University of Alberta, Edmonton, Alberta T6G 1H9, Canada.; ^2^ Heavy Machinery Engineering Research Center of Education Ministry, Taiyuan University of Science and Technology, Taiyuan 030024, China.; ^3^ Cancer Center, The Fifth Affiliated Hospital, Guangzhou Medical University, Guangzhou 510700, China.; ^4^ Department of Chemical Engineering, Tsinghua University, Beijing 10084, China.

## Abstract

Improving the adsorption efficiency of porous adsorbent materials for organic liquids with high viscosity is crucial for addressing oil spill incidents. In this study, a high-performance aerogel adsorbent composed of polyimide (PI), hydroxyapatite nanowires (HAPnws), and reduced graphene oxide (rGO) has been fabricated, which leverages reduced flow tortuosity through anisotropic structures and solar-assisted viscosity reduction via photothermal materials. The prepared anisotropic PI/HAP/rGO aerogel, with directional channels, shows unique mechanical properties with high stiffness along the axial direction and compressibility along the radial direction. PI/HAP/rGO, featuring vertically aligned channels, demonstrated superior adsorption efficiency (the adsorption coefficient *K*_s_ reached 0.37 kg m^−1^ s^−1/2^ for an engine oil with a viscosity of ~144 mPa·s) for oil of varying viscosities compared to similar aerogels with uniform pores, because of the substantially reduced flow tortuosity. The photothermal properties of rGO further enhance the adsorption speed of PI/HAP/rGO for viscous oil under sunlight, including crude oil with ultrahigh viscosity. In addition, PI/HAP/rGO exhibits excellent fire resistance, allowing for reusability via both adsorption–compression and adsorption–combustion cycles. The robust and compressible PI/HAP/rGO aerogels with high adsorption efficiency for viscous oil and fire resistance represent an ideal solution for practical oil spill treatment, and this approach also offers inspiration for the development of advanced adsorbent materials.

## Introduction

Oil contamination from various human activities (oil spills from oil production, transport, shipping, and storage) and natural events (natural seepage) have caused serious impacts on the environment, ecosystems, and economies [1,2]. Researchers have proposed different methods to treat oil spills, such as boom systems [3], in situ burning [4], bioremediation [5], and recovery by adsorbent materials [6]. Porous adsorbent materials, such as foams [7], aerogels [8,9], and sponges [10] with superhydrophobicity and compressibility, have been widely applied to treat oil spills due to their high adsorption efficiency, selective adsorption, reusability, and minimal environmental impact [11]. The driving force for the oil adsorption capacity of the adsorbent materials derives from the van der Waals force and the capillary rise effect [12,13]. Therefore, the study of adsorbent materials for oil spill recovery mainly focuses on hydrophobic surface modification, pore size control, and enhancing compressibility. However, enhancing the adsorption efficiency of adsorbent materials toward oil with high viscosity is still a challenge for researchers.

For most porous adsorbent materials, the randomly distributed pores exert high resistance to the oil, especially for highly viscous oil, to flow into the internal space of the adsorbent materials, resulting in low adsorption capacity and adsorption speed [14]. Porous material with aligned channels should be a better candidate to reduce the tortuosity of the porous adsorbent materials and fully utilize the capillary force. One typical example is the natural wood material, which possesses aligned channels for water transport. Wu and coworkers [15] reported the application of hydrophobically modified wood carbon sponges for fast adsorption and recovery of crude oil via the Joule-heating and photothermal effect. Nevertheless, the adsorption efficiency is limited owing to the low porosity and uncontrollable pore size of the wood sponge. Another approach is fabricating porous materials with aligned channels via directional freeze casting, namely, the ice template method [16–18]. Liu et al. [14] prepared hydrophobic plastic foam with aligned channels via an organic solvent freeze-casting strategy, which showed high adsorption speed and adsorption capacity for oil with a wide range of viscosity. Apart from structural optimization, reducing the oil viscosity via solar energy is also a promising approach to improve the performance of adsorbent materials for highly viscous oil [9,19,20]. Typical photothermal conversion materials include reduced graphene oxides (rGOs) [21], carbon nanotubes [22], and MXene [23]. These materials are usually coated on the adsorbent materials to increase temperature under the sunlight, which reduces the oil viscosity and accelerates the adsorption speed. Therefore, it would be a promising approach to combine the above 2 strategies, namely, aligned channel structure and solar assistant viscosity reduction, in the preparation of efficient adsorbent materials for highly viscous oil.

To fabricate such porous adsorbent materials, polyimide (PI) was chosen as the polymer component due to its excellent thermal stability, high mechanical strength, and hydrophobicity after the thermal imidization process [24–28]. However, the PI aerogels suffer from severe shrinking problems during the thermal imidization process, which makes it difficult to control the pore size and porosity of the PI aerogel. Incorporating nanomaterials, including nanoparticles, nanowires, and nanosheets, into PI aerogels could effectively reduce the shrinking degree [29,30]. Compared with the particulate additives, the fibrillar nanowires and flaky nanosheets have stronger interaction and entanglement with the PI chains, which usually leads to more notable shrinkage reduction [31–34]. Hydroxyapatite nanowires (HAPnws) are novel one-dimensional nanomaterials that have been widely studied for the fabrication of composite materials due to their high mechanical strength, excellent thermal stability, and great compatibility with organic materials [35–37]. Therefore, HAPnws were selected as the inorganic phase in the composite aerogel to enhance its mechanical and thermal properties. Various photothermal conversion materials, such as rGO [38], carbon nanotubes [39], MXene [24], and polypyrrole [40], have been incorporated with PI to fabricate functional aerogels for solar evaporation. The hydrophilic GO can be thermally reduced to hydrophobic rGO by eliminating the oxygen-contained groups in GO, which makes the rGO an ideal building material for fabricating adsorbent materials for hydrophobic organic liquids [41].

Herein, incorporating the PI polymer, HAPnws, and rGO, a high-performance anisotropic adsorbent for viscous oil was prepared via directional freeze casting. The embedding of HAPnws in the PI chain network and the coverage of rGO nanosheet on PI polymer greatly enhanced the mechanical strength of the prepared PI/HAP/rGO aerogel and markedly reduced its shrinking rate. The anisotropic structure of PI/HAP/rGO endowed its high toughness along the axial direction and compressibility along the radial direction. Moreover, the intrinsic hydrophobicity of PI polymer and rGO imparted PI/HAP/rGO aerogel the selective adsorption of organic liquid. PI/HAP/rGO exhibited excellent adsorption efficiency for organic liquid with various viscosity, and the adsorption efficiency could be further improved by the illumination of sunlight. Meanwhile, the unique mechanical property and remarkable fire resistance of PI/HAP/rGO guaranteed its reusability via both adsorption–compression and adsorption–combustion cycles. Therefore, this study provides new insights into the design of porous adsorbent for highly viscous oil, and the prepared PI/HAP/rGO aerogel has great application potential for fast cleaning and recovery of viscous oil spills.

## Results and Discussion

### Synthesis and characterization of materials

**Fig. 1.**
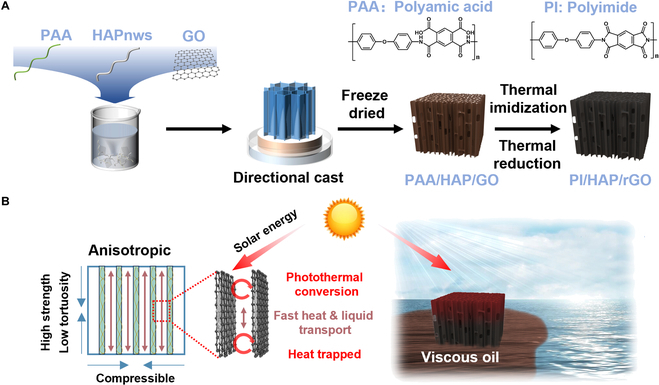
(A) Schematic of the synthesis route of the PI/HAP/rGO aerogel. (B) Illustration of the structure, properties, and application of the PI/HAP/rGO aerogel.

PI/HAP/rGO aerogel was fabricated by directional freeze casting of the polyamic acid (PAA), HAPnws, and GO mixture, followed by freeze-drying and thermal treatment, as shown in Fig. 1A. For the aerogel prepared with different HAPnw contents, samples were named in the format of PI/HAP*x*/rGO (*x* = 0.3, 0.6, 0.9, 1.2, and 1.5, indicating the concentration of HAPnws) (Fig. S1). Samples prepared with 1 or 2 components were labeled using the abbreviations of those components, such as PI, PI/rGO, and PI/HAP*x* (*x* = 0.3, 0.6, 0.9, 1.2, and 1.5, indicating the concentration of HAPnws). The obtained aerogel is hydrophobic due to the thermal reduction of GO to rGO and the imidization of PAA to PI. Additionally, the anisotropic structure of PI/HAP/rGO results in low tortuosity along the vertical direction, which could achieve fast heat and liquid transport for the application of solar-assisted adsorption of organic liquid with high viscosity, including heavy crude oil (Fig. 1B).

**Fig. 2.**
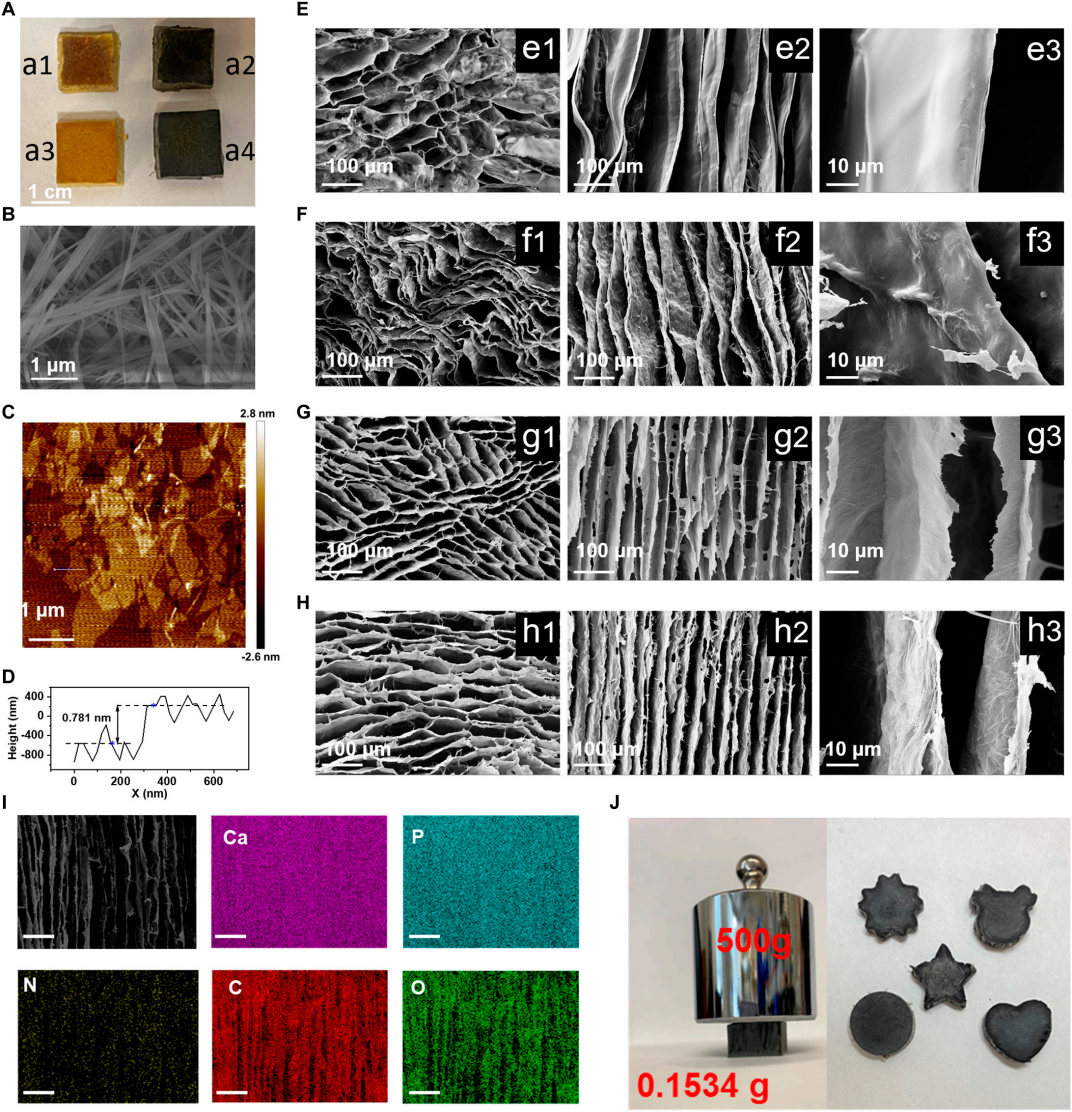
Morphology characterization of PI/HAP/rGO aerogel and its components. (A) Photos of aerogels with different compositions (A1) PI, (A2) PI/rGO, (A3) PI/HAP0.9, and (A4) PI/HAP0.9/rGO. (B) SEM image of HAPnws. (C) AFM image of GO. (D) Thickness measurement of GO. SEM images of (E) PI, (F) PI/rGO, (G) PI/HAP0.9, and (H) PI/HAP0.9/rGO along (E1, F1, G1, H1) radial direction, (E2, F2, G2, H2) axial direction, and (E3, F3, G3, H3) axial direction at high magnification. (I) SEM-EDX mapping images of PI/HAP0.9/rGO. Scale bar, 100 μm. (J) Photos of the vertical bearing test of PI/HAP0.9/rGO and PI/HAP0.9/rGO with various shapes.

Figure 2A shows the photos of PI, PI/rGO, PI/HAP0.9, and PI/HAP0.9/rGO aerogels. PI and PI/HAP0.9 aerogels present a yellow appearance, while PI/rGO and PI/HAP0.9/rGO show a black color due to the existence of rGO. The scanning electron microscope (SEM) image of HAPnws shows their fibrous structure with a length of tens of micrometers in the form of nanowire bundles (Fig. 2B). The atomic force microscope (AFM) image of GO indicates that the literal size of the synthesized GO is in the range of hundreds of nanometers to micrometers (Fig. 2C). Based on the AFM image, the thickness of GO is measured to be ~0.781 nm (Fig. 2D). The SEM images of PI, PI/rGO, PI/HAP, and PI/HAP/rGO aerogels are presented in Fig. 2E to H. Since all these aerogels are fabricated via the directional casting method, the vertically aligned channels are observed in SEM images along the axial direction of all 4 kinds of aerogels. PI and PI/GO aerogels exhibit relatively large and irregular pores at the transverse section, and wider channels with soft and bent walls are observed along the axial direction (Fig. 2E1, E2, F1, and F2). However, both PI/HAP and PI/HAP/rGO aerogels have regular lamellar structures and smaller pore sizes at the transverse section, and the channel size is smaller with rigid and straight walls as shown in the longitudinal section images (Fig. 2G1, G2, H1, and H2). The SEM images of PI/HAP and PI/HAP/rGO with higher magnification show that the HAPnws are entangled with each other and embedded in the PI polymers, which dramatically improves the structural stability of these aerogels during the fabrication process (Fig. 2G3 and H3). Comparing the wall surface of PI and PI/rGO aerogels (Fig. 2E3 and F3), the wall surface of PI aerogel is perfectly smooth, but wrinkles are observed on the wall surface of PI/rGO, which indicates that rGO sheets cover the PI polymer. The energy-dispersive x-ray spectroscopy element (EDX) mapping images of PI/HAP/rGO show that Ca, P, N, C, and O elements are uniformly distributed, reflecting the great dispersibility of HAPnws and GO nanosheet in the PAA solution. With the vertically aligned structure and the interwoven network between the HAPnws and PI polymer chains, PI/HAP/rGO aerogels have excellent mechanical strength along the vertical direction. As shown in Fig. 2J, a cubic PI/HAP/rGO aerogel weighing 0.1534 g withstood a weight of 500 g in the vertical direction, which is ~3,259 times its own weight. More detailed discussions on the mechanical properties will be presented in later sections. By changing the casting mold, the shape and size of PI/HAP/rGO could be easily regulated, such as shapes of flower, bear, pentagram, round, and heart (Fig. 2J).

**Fig. 3.**
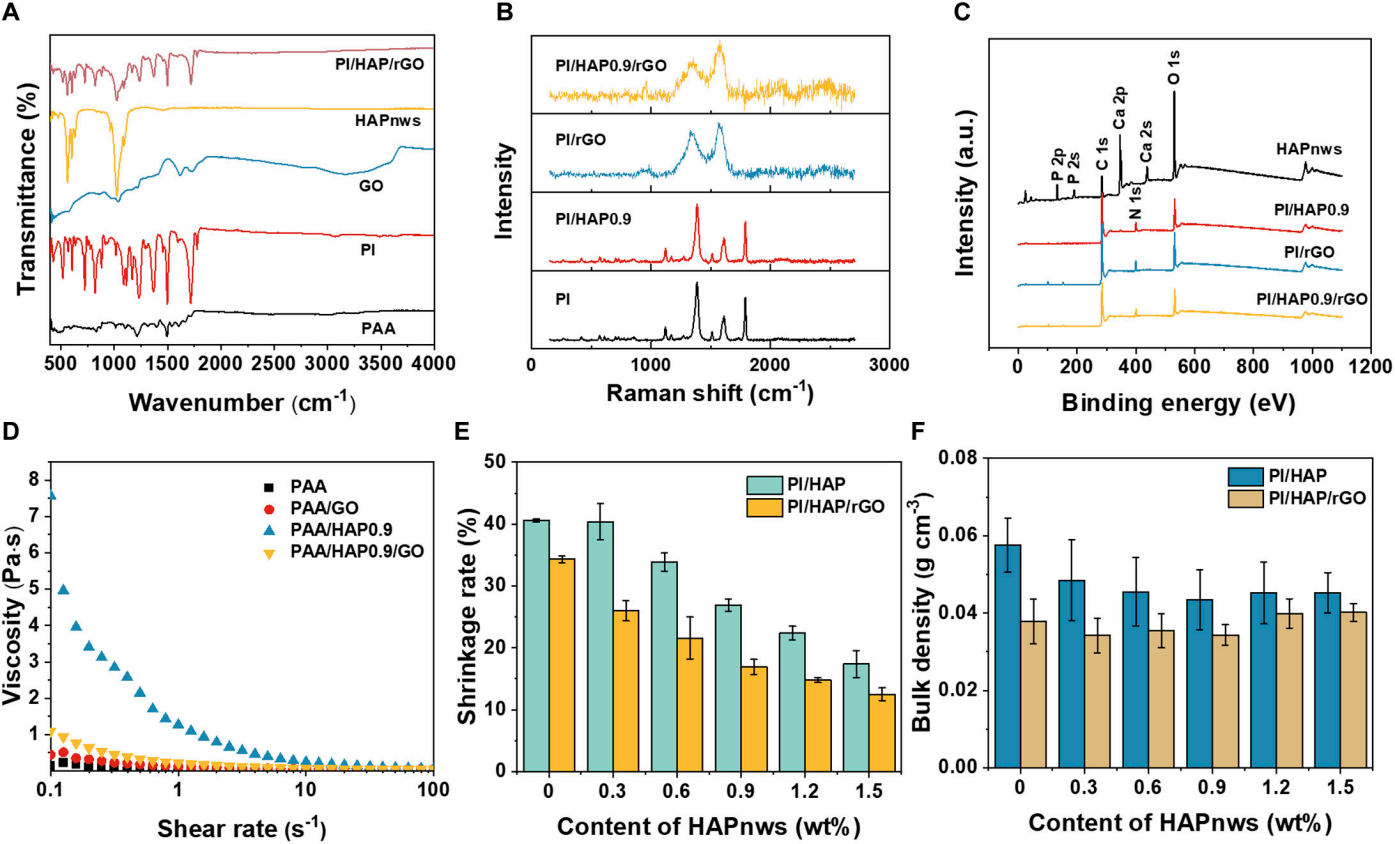
Chemical characterization and physical properties of PI/HAP/rGO. (A) FTIR spectra of PAA, PI, GO, HAPnws, and PI/HAP/rGO. (B) Raman spectra of PI, PI/HAP0.9, PI/rGO, and PI/HAP0.9/rGO. (C) XPS survey scan of HAPnws, PI/HAP0.9, PI/rGO, and PI/HAP/rGO. (D) Rheological properties of PAA, PAA/GO, PAA/HAP0.9, and PAA/HAP0.9/GO solutions. (E) Shrinking rate of PI/HAP and PI/HAP/rGO with different contents of HAPnws. (F) Density of PI/HAP and PI/HAP/rGO with different contents of HAPnws.

The chemical composition of the prepared composite aerogel was analyzed by Fourier transform infrared spectroscopy (FTIR), Raman spectrum, and x-ray photoelectron spectroscopy (XPS). Figure 3A shows the FTIR spectrum of PI/HAP0.9/rGO and the spectra of its components. Compared with the spectrum of PAA, the new peaks at 1,718 and 1,775 cm^−1^, corresponding to the asymmetric and symmetric stretching vibrations of carbonyl in imide rings, indicate the successful thermal imidization of PAA to PI [42]. Peaks at 1,095, 1,026, and 961 cm^−1^ are the characteristic peaks of PO_4_^3−^ in HAPnws [37]. The spectrum of PI/HAP0.9/rGO includes all characteristic peaks of PI, HAPnws, and rGO. Pure PI aerogel and PI/HAP0.9 aerogel almost present the same Raman spectrum, which indicates that the HAPnws are embedded in the PI polymer network to act as the “bones” of the composite aerogel [43]. The Raman spectra of PI/rGO and the same aerogel before the thermal treatment (PAA/GO) are presented in Fig. S2A. After the thermal reduction process, the *I*_D_/*I*_G_ ratio of PAA/GO decreased from 1.91 to 1.45 of PI/rGO. The much lower *I*_D_/*I*_G_ ratio in PI/rGO reflects a higher content of sp2-hybridized carbon atoms in the rGO (Fig. S2A). After introducing rGO into the system, both PI/rGO and PI/HAP/rGO exhibit only 2 peaks of the D band and G band for rGO, reflecting that the outer layer of the aerogels is covered by rGO nanosheets (Fig. 3B). XPS results further verified the structure of PI/HAP/rGO from the surface chemical composition aspect. As shown in Fig. 3C, the XPS curve of HAPnws exhibits the characterization peaks of Ca 2s (438.96 eV), Ca 2p (348 eV), P 2s (188.86 eV), and P 2p (135 eV), while only C 1s (284.8 eV), N 1s (400.6 eV), and O 1s (532.6 eV) are observed in the XPS curves of PI/HAP0.9, PI/rGO, and PI/HAP0.9/rGO. Meanwhile, the intensity of N 1s peak in PI/HAP0.9/rGO is smaller than that in PI/HAP. Based on the results of SEM images, Raman, and XPS, it could be concluded that HAPnws were embedded in PI and rGO nanosheets covered on the PI surface. The high-resolution XPS spectra of C 1s and O 1s of PI/HAP0.9/rGO are presented in Fig. S2B and C, in which the fitted peaks are mainly attributed to the chemical bonds in rGO. The interaction among PAA, HAPnws, and GO was also investigated via the rheological behavior (Fig. 3D). The additive of GO only slightly enhanced the viscosity of PAA solutions, while the addition of 0.9 wt% HAPnws considerably increased the viscosity of PAA solution from 0.139 to 0.656 Pa·s, which should be ascribed to the strong interaction between the Ca^2+^ on HAPnw surface and carboxyl groups on PAA chain. However, the presence of GO in the PAA/HAP system decreased the viscosity, indicating that the GO nanosheet may weaken the interaction between PAA and HAPnws. The content of HAPnws in the PI/HAP/rGO aerogel plays an important role in the shrinkage rate and density of the composite aerogel. The photo of PI/HAP and PI/HAP/rGO aerogels with different HAPnw content is shown in Fig. S1. Aerogels with lower HAPnw content show smaller sizes. As shown in Fig. 3E, the shrinkage rate of PI/HAP and PI/HAP/rGO aerogels decreases gradually with the increasing content of HAPnws. The shrinkage of composite aerogels results from the imidization of PAA to PI, which causes a shrinkage rate of 40.54% of pure PI aerogel. After the addition of 1.5 wt% HAPnws, the shrinkage rate of PI/HAP1.5 is reduced to 17.39%. Meanwhile, PI/HAP/rGO aerogels have lower shrinkage rates than PI/HAP aerogels at the same HAPnw content. Both HAPnws and rGO support the PI polymer network to reduce its shrinkage during imidization. SEM images in Figs. S3 to S5 show that PI/HAP/rGO with higher content of HAPnws exhibits more rigid and regular structures, which enhances the structure stability of the composite aerogel. The higher content of HAPnws decreased the shrinkage but also increased the solid composition. Therefore, the lowest density is achieved by PI/HAP0.9/rGO, with a density of 0.034 g cm^−3^ (Fig. 3F). Figure S2D shows the N_2_ adsorption–desorption isotherms of PI/HAP0.9/rGO aerogel, and the surface area was calculated to be 13.998 m^2^/g. This relatively small surface area indicates that the vertical channels, with a width of 20 to 30 μm and a length in the centimeter scale, are the primary pores in the PI/HAP0.9/rGO aerogel.

### Mechanical property of the composite aerogel

**Fig. 4.**
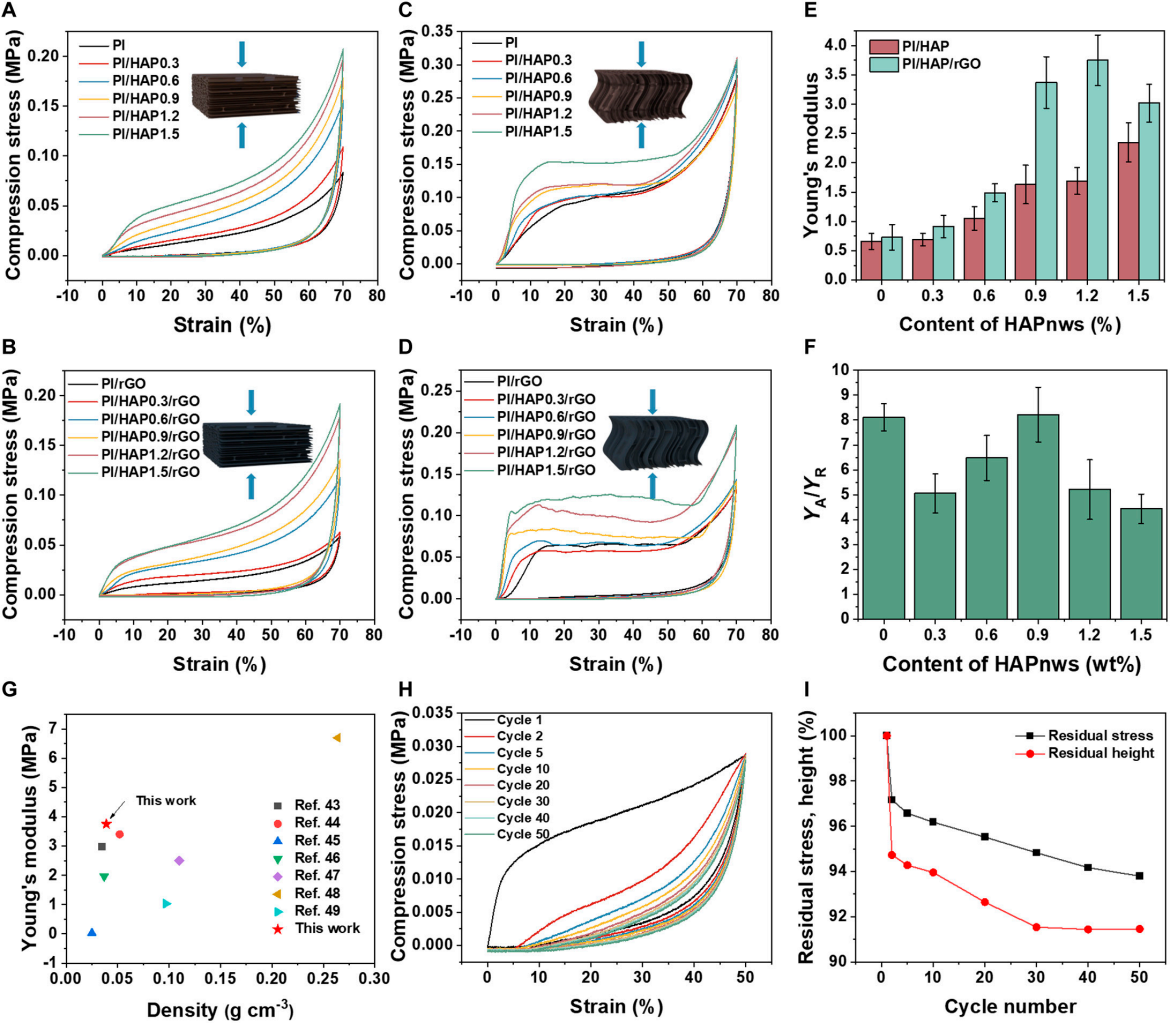
Mechanical properties of PI/HAP and PI/HAP/rGO. Compressive stress–strain curves of (A) PI/HAP and (B) PI/HAP/rGO with different contents of HAPnws along the radial direction. Compressive stress–strain curves of (C) PI/HAP and (D) PI/HAP/rGO with different contents of HAPnws along the axial direction. (E) Young’s modulus of PI/HAP and PI/HAP/rGO along the axial direction with different contents of HAPnws. (F) Ratio of *Y*_A_/*Y*_R_ of PI/HAP/rGO with different contents of HAPnws. (G) Comparison of Young’s modulus of PI/HAP/rGO with that of aerogels in previous reports [43,46–51]. (H) Fifty times compressive fatigue test of PI/HAP/rGO along the radial direction. (I) Variation of residual stress and residual height of the PI/HAP/rGO aerogel during the cyclic compressive test.

The mechanical properties of PI/HAP and PI/HAP/rGO composite aerogels with different contents of HAPnws were investigated in both the axial and radial directions (Fig. 4). Due to the anisotropic structure, the composite aerogels exhibit completely different mechanical properties in different directions. As shown in Fig. 4A and B, both PI/HAP and PI/HAP/rGO aerogels show elasticity along the radial direction. For pure PI aerogel, the stress–strain curve shows a linear relationship due to the gradual densification, while the slope increases sharply in the late deformation stage because of the greatly increased density. With the increasing content of HAPnws, the stress–strain curves tend to present a trend with 3 stages. Take PI/HAP1.5 as an example, the stress increases linearly with a relatively large slope at strain less than 10%, which is ascribed to the stiffness provided by the HAPnws. Then, the slope decreased slightly because the gradual densification dominates in this stage. The slope in the last stage increases faster due to the extremely increased density at such a high strain. PI/HAP/rGO exhibits similar stress–strain curves, but the slope in the first stage is higher than that of PI/HAP because the rGO nanosheets covered on the matrix surface further increase the aerogel’s stiffness. The stress–strain curves of PI/HAP and PI/HAP/rGO along the axial direction show different patterns from the radial direction in the second stage during compression (Fig. 4C and D). After the first elastic region, the aerogels yield and undergo plastic deformation, at which stage the stress is almost unchanged with increasing strain. Then, further irreversible densification resulted in a sharp increase in stress with strain. The slope of the stress–strain curve at the first stage gives Young’s modulus of the aerogel along individual directions. Both PI/HAP and PI/HAP/rGO aerogels have larger Young’s modulus along the axial direction (*Y*_A_) than radial direction (*Y*_R_). *Y*_A_ of PI/HAP and PI/HAP/rGO and *Y*_A_/*Y*_R_ of PI/HAP/rGO are summarized in Fig. 4E and F, respectively. *Y*_A_ values of PI/HAP/rGO are larger than those of PI/HAP at the same HAPnw content, and higher HAPnw content achieves higher *Y*_A_ in a certain range. The *Y*_A_ values of PI/HAP0.9/rGO and PI/HAP1.2/rGO reach 3.37 and 3.75 MPa, respectively. However, further increasing HAPnw content to 1.5 wt% reduces the *Y*_A_ to 3.02 MPa, which may be ascribed to the aggregates of GO when excessive HAPnws are introduced as shown in Fig. S5. PI/HAP0.9/rGO exhibit the largest *Y*_A_/*Y*_R_, indicating that PI/HAP0.9/rGO is highly robust along the axial direction while easy to be compressed along the radial direction. The mechanical properties of PI/HAP0.9/rGO with uniform pore (UP) structure (Fig. S6) and composite aerogel prepared with hydroxyapatite nanoparticles at the same concentrations of each component (PI/HAP-NP/rGO) were also tested (Fig. S7). PI/HAP0.9/rGO with UP structure shows similar stress–strain curves along the axial direction and radial direction, with a much smaller Young’s modulus (~0.15 MPa). For the system of PI/HAP-NP/rGO with directional channel (DC) structure, the stress–strain curves along 2 directions have a similar pattern with PI/HAP0.9/rGO. However, Young’s modulus of PI/HAP-NP/rGO along 2 directions (0.81 MPa along the axial direction and 0.15 MPa along the radial direction) is much smaller than that of PI/HAP0.9/rGO. These results demonstrate the advantages of HAPnws and the DC structure in improving the mechanical properties of the composite aerogels. Compared with other aerogels in previous reports, PI/HAP0.9/rGO shows a relatively high Young’s modulus with extremely low density, which gives it great application potential in various industries (Fig. 4G). To further explore the compressibility of PI/HAP0.9/rGO along the radial direction, a 50 times compressive fatigue test with a constant strain of 50% was performed. As shown in Fig. 4H and I, although the loading–unloading loop decreases dramatically from the first compression to the second compression, and then gradually decreases over the 50 times compression, the residual stress and residual height are still higher than 93.79% and 91.46% respectively.

### Oil adsorption property of PI/HAP0.9/rGO

**Fig. 5.**
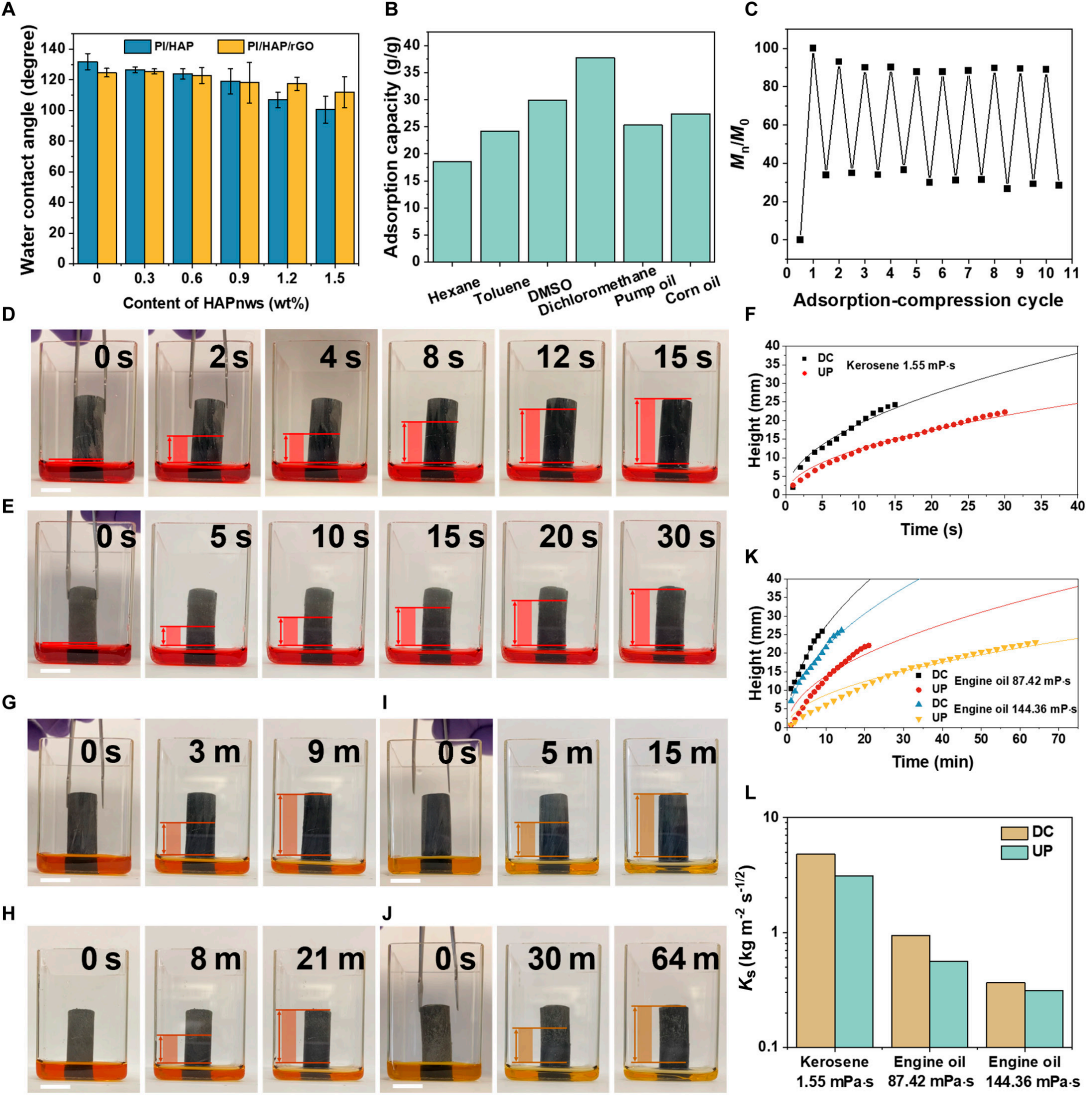
Adsorption properties of PI/HAP0.9/rGO for organic liquids with different viscosity. (A) Water contact angle of PI/HAP/rGO aerogel with different contents of HAPnws. (B) Adsorption capacity of PI/HAP0.9/rGO for different organic liquids. (C) Adsorption recyclability of PI/HAP0.9/rGO for corn oil within 10 times adsorption–compression cycles. (D) Chronological photos of the adsorption process of PI/HAP0.9/rGO with DC structure for kerosene. Scale bar, 1 cm. (E) Chronological photos of the adsorption process of PI/HAP0.9/rGO with UP structure for kerosene. Scale bar, 1 cm. (F) Chronological photos of the adsorption process of PI/HAP0.9/rGO with DC structure for engine oil-L. (G) Chronological photos of the adsorption process of PI/HAP0.9/rGO with UP structure for engine oil-L. Scale bar, 1 cm. (H) Chronological photos of the adsorption process of PI/HAP0.9/rGO with DC structure for engine oil-H. Scale bar, 1 cm. (I) Chronological photos of the adsorption process of PI/HAP0.9/rGO with UP structure for engine oil-H. (J) Kerosene adsorption height–time curves of PI/HAP0.9/rGO with DC and UP structures. (K) Engine oil adsorption height–time curves of PI/HAP0.9/rGO with DC and UP structures. (L) Kerosene and engine oil adsorption coefficients of PI/HAP0.9/rGO with DC and UP structures.

During the thermal treatment, the imidization and reduction of GO increased the hydrophobicity of the composite aerogels. As shown in Fig. 5A, the water contact angle of pure PI aerogel reaches 131.9°. With the increasing content of HAPnws (superhydrophilic), the water contact angle gradually decreases to 100.53° for PI/HAP1.5. For the system of PI/HAP/rGO, the effect of HAPnws is similar but less important owing to the coverage of rGO. The water contact angle decreases from 124.7° to 111.94° as HAPnw content increases from 0 to 1.5 wt%. Previous results have shown the excellent mechanical properties of PI/HAP0.9/rGO. Furthermore, the water contact angle of PI/HAP0.9/rGO is around 118.17°. Therefore, PI/HAP0.9/rGO was selected for the following oil adsorption tests. PI/HAP0.9/rGO exhibits high adsorption capacity toward various organic solvents and oils. The adsorption capacity ranges from 18.61 to 37.77 g/g, depending on the density of the oil. Due to the highly compressible properties of PI/HAP0.9/rGO, the adsorption–compression cycle test was performed using corn oil as the target oil. After 10 cycles, the adsorption capacity remains at 88.92% of the initial adsorption capacity, indicating the great reusability of PI/HAP0.9/rGO as a waste oil adsorbent. More importantly, the vertically aligned channels of PI/HAP0.9/rGO endow the aerogel with exceptional adsorption speed. The adsorption speed of aerogel is mainly affected by the viscosity of oil. Kerosene with a viscosity of 0.8 mPa·s was applied as the model oil for low-viscosity oil. As depicted in Fig. 5D, E, and J, PI/HAP0.9/rGO with DCs achieves an adsorption height of 24.20 mm within 15 s, while PI/HAP0.9/rGO with UPs requires 30 s to realize an adsorption height of 22.26 mm. Furthermore, 2 different kinds of engine oil with viscosity of 87.42 mPa·s (engine oil-L) and 144.36 mPa·s (engine oil-H) were applied for the adsorption speed test. The increasing viscosity of oil dramatically reduced the adsorption speed of aerogels. Figure 5G, H, and K shows that DC and UP take 9 and 21 min to reach the adsorption height of 25.92 mm and 22.09 mm, respectively, for the engine oil-L. Further increasing the viscosity, UP costs more than 4 times as much time as the DC needs to reach full adsorption (Fig. 5I, J, and K). Figure 5F and K records the curves of adsorption height versus time for oil with different viscosities. According to Washburn et al. [44], the adsorption liquid mass per unit area (*m_u_*) has a linear relationship with the adsorption time^½^ (*t*^½^), which can be approximately fitted with the following equations:

$$m_{u}=K_{s}t^{1/2}$$ (1)

$$m_{u}=\mathrm{H\rho}$$ (2)

where *H* means the adsorption height, *ρ* means the density of the oil, and *K*_s_ means the liquid adsorption coefficient. By fitting the *m_u_*–*t*^½^ curves with Eq. 1, the *K*_s_ values of DC and UP for oil with various viscosities were obtained (Fig. S8 and Fig. 5L). The *K*_s_ values of DC are larger than those of UP for all 3 types of oil, reflecting the considerably enhanced adsorption speed of PI/HAP/rGO with the DC structure. *K*_s_ can also be defined by Eq. 3:

$$K_{s}=\rho {\left(\frac{\sigma }{\mu }\right)}^{1/2}\left(\frac{\varepsilon^{*}}{\tau }\right){r_{0}}^{1/2}{\left(\text{cos}\frac{\theta }{2}\right)}^{1/2}$$ (3)

where *ρ* is the liquid density, *μ* is the liquid viscosity, *σ* is the liquid’s surface tension, ε* is the effective absorption porosity, *τ* is the average tortuosity factor, *r*_0_ is the average channel radius, and *θ* is the contact angle between the adsorbed liquid and the adsorbent material. For a certain oil, PI/HAP0.9/rGO with DC and UP structures share the same *ρ*, *μ*, *σ*, and *θ*. Based on previous discussions, DC and UP also have similar porosity and SEM images show that they have comparable *r*_0_ (Fig. S6). Therefore, *τ* dominates the value of *K*_s_, and PI/HAP0.9/rGO with the DC structure greatly reduces the tortuosity of liquid transportation in the aerogel to achieve faster adsorption capacity.

### Solar-assisted oil adsorption property of PI/HAP0.9/rGO

**Fig. 6.**
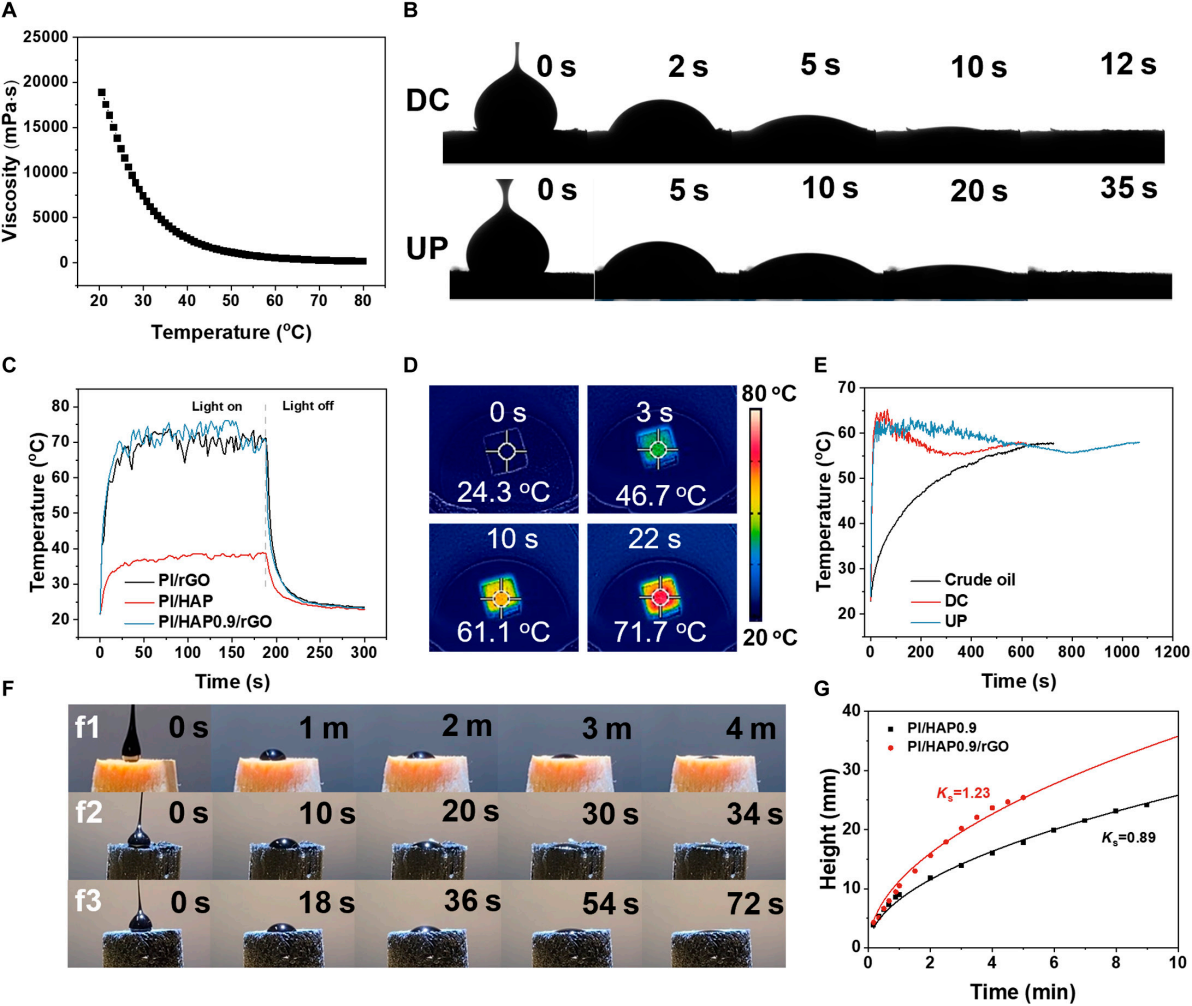
Viscous oil adsorption test of PI/HAP0.9/rGO under simulated sunlight. (A) Viscosity of crude oil changed with temperature. (B) Adsorption process of a crude oil droplet into PI/HAP0.9/rGO with DC and UP structures. (C) Surface temperature of PI/rGO, PI/HAP0.9, and PI/HAP0.9/rGO aerogel as a function of time under one sun illumination. (D) Infrared images of PI/HAP0.9/rGO at different illumination time. (E) Surface temperature of PI/HAP0.9/rGO with DC and UP structures floated on crude oil, and crude oil under one sun illumination. (F) Adsorption process of a crude oil droplet into the (F1) PI/HAP0.9, (F2) PI/HAP0.9/rGO with DC, and (F3) UP structure. (G) Adsorption height–time curves of PI/HAP0.9 and PI/HAP0.9/rGO toward engine oil-L under one sun illumination.

The results in previous sections verified that PI/HAP0.9/rGO achieved faster adsorption for oil with lower viscosity. Meanwhile, the viscosity of a liquid usually reduces with the increase in temperature. Therefore, the oil adsorption performance of PI/HAP0.9/rGO aerogel can be further improved with the assistance of sunlight since rGO is recognized as an excellent photothermal conversion material. Crude oil is a typical liquid for which viscosity decreases with increasing temperature. So, the crude oil from the Athabasca oilfield was used for the following tests. Figure 6A shows that crude oil has a high viscosity at 20 °C, while increasing the temperature to 60 °C remarkably reduces its viscosity. The adsorption process of a crude oil droplet on PI/HAP0.9/rGO aerogel with DC and UP structures at 60 °C is presented in Fig. 6B. For the DC structure, the crude oil droplet was adsorbed completely within 12 s. However, PI/HAP0.9/rGO with the UP structure took 35 s to fully adsorb the crude oil droplet, which further confirmed the low tortuosity of the DC structure for liquid adsorption. Figure 6C exhibits that the surface temperature of PI/HAP0.9/rGO and PI/rGO rises to about 70 °C within 50 s under one sun illumination (1 kW/m^2^), while the one sun illumination can only increase the surface temperature of PI/HAP0.9 to ~38 °C. The infrared images of PI/HAP0.9/rGO during the illumination process are shown in Fig. 6D. After being illuminated for only 22 s, the surface temperature of PI/HAP0.9/rGO rose to 71.7 °C, which was ascribed to the excellent photothermal conversion properties of rGO. Under one sun illumination, the surface temperature of crude oil, and PI/HAP0.9/rGO with DC and UP structures (with a height of 0.5 cm) floating on crude oil, is recorded in Fig. 6E. Crude oil could be gradually heated to ~58 °C within 750 s. The surface temperature of PI/HAP0.9/rGO with DC and UP structures both instantly rose to over 60 °C within 50 s. Then, PI/HAP0.9/rGO with the DC structure began to decrease at ~100 s, which means that the crude oil was adsorbed into the aerogel and the oil surface was approaching the aerogel’s top surface. The lowest temperature was observed at ~350 s, indicating the time that crude oil reached the top surface and then the crude oil on the top surface was gradually heated to the temperature that the crude oil could reach under one sun illumination. Based on the height and density of the crude oil, the adsorption coefficient of PI/HAP0.9/rGO for crude oil could be calculated to be 0.25 kg m^−2^ s^−1/2^. For PI/HAP0.9/rGO with the UP structure, the surface temperature began to drop at ~350 s and then gradually decreased to around 56 °C at ~800 s. After that, the surface temperature progressively rose to ~58 °C. The much later surface temperature drops for PI/HAP0.9/rGO with the UP structure than that for the one with the DC structure indicated the faster adsorption of crude oil in the DC structure under solar-assisted situation, which should be not only ascribed to the low tortuosity of the vertically aligned channel but also due to the faster heat transfer along the DC. Figure 6F further exhibits the adsorption process of a crude oil droplet into PI/HAP0.9, and PI/HAP0.9/rGO aerogel with DC and UP structures under one sun illumination. With the assistance of sunlight, PI/HAP0.9 took 4 min to fully adsorb the crude oil droplet. Surprisingly, the oil droplet was adsorbed into the aerogel within 34 and 72 s for PI/HAP0.9/rGO with DC and UP structures, respectively, which verified the importance of both anisotropic structure and the photothermal thermogenesis property of rGO in improving the adsorption efficiency of highly viscous crude oil. For other viscous oils whose viscosity is not as sensitive as crude oil to temperature change, PI/HAP0.9/rGO also presented improved adsorption efficiency with the assistance of sunlight. Figure S9 shows that the viscosity of engine oil-L was reduced from 87.42 mPa·s at 25 °C to 8.53 mPa·s at 80 °C. As shown in Fig. 6G, it cost 9 min for PI/HAP0.9 to reach an adsorption height of 24.10 mm for the adsorption of engine oil-L, which exhibited a compatible adsorption coefficient (0.89 kg m^−2^ s^−1/2^) with PI/HAP0.9/rGO without the assistance of sunlight. However, under the illumination of sunlight, PI/HAP0.9/rGO achieved an adsorption coefficient of 1.23 kg m^−2^ s^−1/2^ for the same engine oil. The *m_u_*–*t*^½^ curves are shown in Fig. S10. Compared with the reported adsorbent in the literature, PI/HAP0.9/rGO presents a superior adsorption coefficient (Table S1).

### Fire-retardant property of PI/HAP0.9/rGO

**Fig. 7.**
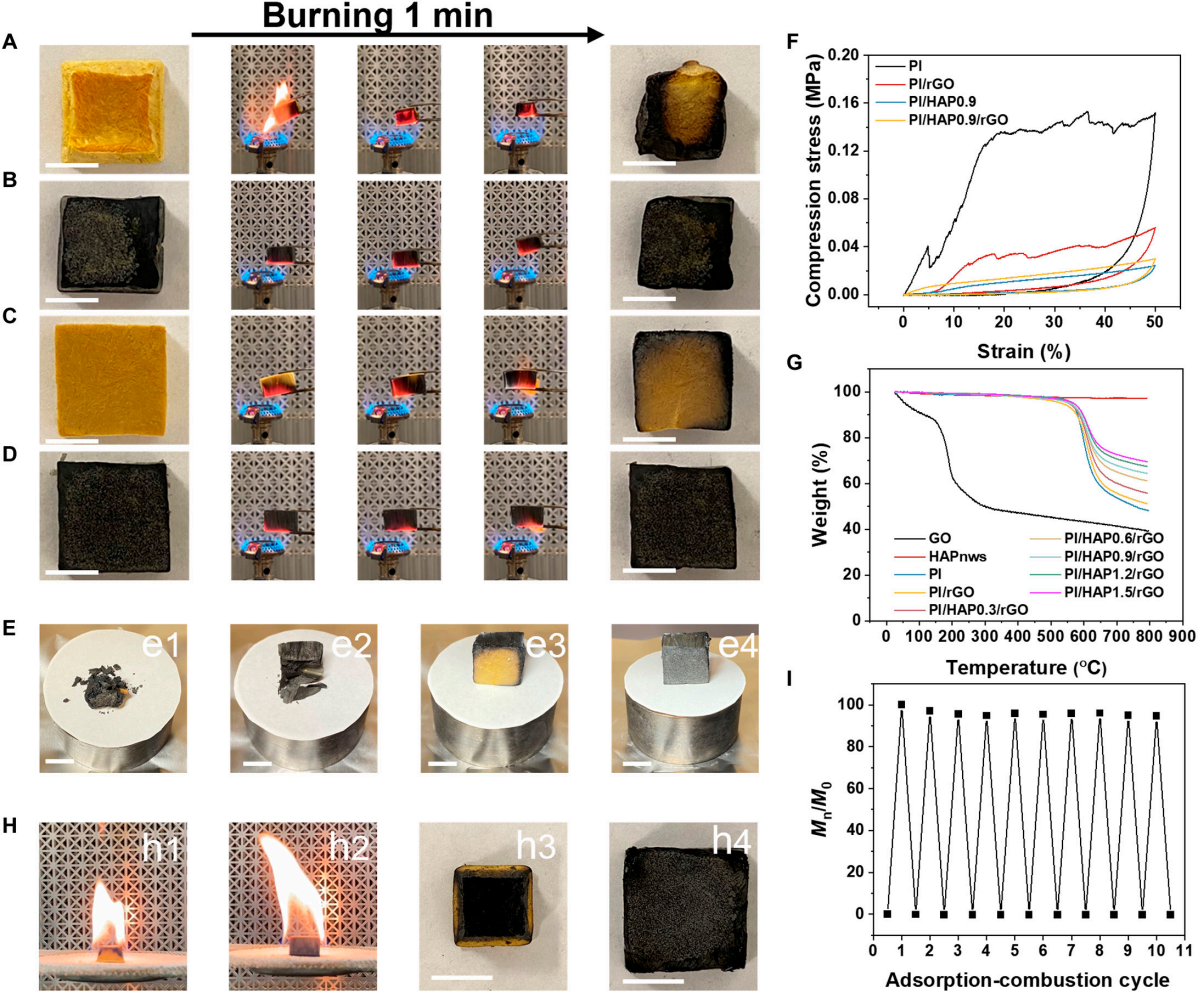
Flame-retardant properties of the PI/HAP/rGO aerogel. Photos before, during, and after the burning of (A) PI, (B) PI/rGO, (C) PI/HAP0.9, and (D) PI/HAP0.9/rGO. (E) Photos of the burned (E1) PI, (E2) PI/rGO, (E3) PI/HAP0.9, and (E4) PI/HAP0.9/rGO after the compressive test along the radial direction. (F) Compressive stress–strain curves of the burned PI, PI/rGO, PI/HAP0.9, and PI/HAP0.9/rGO. (G) TGA curves of PI/HAP/rGO with different contents of HAPnws and the curves of individual components. (H) Photos of burning (H1) PI and (H2) PI/HAP0.9/rGO adsorbed with hexane and photos of the burned (H3) PI and (H4) PI/HAP/rGO. (I) Adsorption–combustion cycle of PI/HAP0.9/rGO for adsorption of hexane. Scale bars, 1 cm.

The reusability of an adsorbent material is critical for its practical application. Previous results verified that the great compressibility of PI/HAP0.9/rGO and the adsorbed oil could be recovered by squeezing the PI/HAP0.9/rGO aerogel along the radial direction. Moreover, PI/HAP0.9/rGO could be reused through combustion due to the great fire resistance of rGO and HAPnws. Figure 7A to D shows the 1-min combustion process of PI, PI/rGO, PI/HAP0.9, and PI/HAP0.9/rGO on a butane torch at different stages. The PI aerogel expanded the flame when it contacted with fire, and the majority of PI aerogel changed from yellow to black after burning for 1 min, which should be ascribed to the pyrolysis of the PI chain (Fig. 7A). Figure 7E1 and F shows the picture of burned PI aerogel after the compression test and the corresponding compression stress–strain curve, reflecting the loss of compressibility of PI aerogel after burning. PI/rGO aerogel presented better fire resistance than PI aerogel, but it still lost its compressibility and was crushed after one cycle of compression test (Fig. 7E2 and F). The presence of HAPnws greatly enhanced the fire resistance of PI/HAP0.9 aerogel. Only the outer part of PI/HAP0.9 became black after the burning process, and the burned PI/HAP0.9 kept its integrity after the compression test (Fig. 7E3). For PI/HAP0.9/rGO, the appearance is almost unchanged except slightly shrank after the 1-min burning. Moreover, the gradually increased stress with increasing strain and the fully recovered shape in the compression test verified the compressibility of the burned PI/HAP0.9/rGO, indicating that PI/HAP0.9/rGO can prevent fire from spreading into its internal space. Thermogravimetric analysis (TGA) test shows that GO decomposed greatly before 300 °C due to the oxygen-contained groups and then slowly decomposed between 300 and 800 °C (Fig. 7G). The weight loss of HAPnws in the TGA test is less than 3 wt%, reflecting its excellent thermal stability. The fast decomposition of PI polymer occurred at ~600 °C. With the incorporation of rGO and HAPnws, the fast decomposition temperature gradually increased, which facilitated the thermal stability of PI/HAP/rGO aerogel during the combustion test. Last, the adsorption–combustion cycle test was performed, using hexane as the flammable organic liquid. As shown in Fig. 7H and Movie S1, the shape of PI/HAP0.9/rGO barely changed after the adsorbed hexane burned out, while PI aerogel shrank and became black after combustion. Figure 7I shows the excellent reusability of PI/HAP0.9/rGO via the adsorption–combustion process, with 94.58% of the adsorption capacity remaining after 10 adsorption–combustion cycles.

## Conclusion

In summary, a PI/HAP/rGO aerogel with unique mechanical properties, intrinsic hydrophobicity, photothermal conversion, and fire resistance was fabricated for fast adsorption of viscous oil with the assistance of solar light. Because of the vertically aligned channels obtained via the directional casting, the PI/HAP0.9/rGO aerogel exhibits high strength along the axial direction and great compressibility along the radial direction simultaneously, with a *Y*_A_/*Y*_R_ over 8. Moreover, the vertically aligned channel of PI/HAP0.9/rGO dramatically reduced the tortuosity for oil adsorption compared with the same aerogel with UPs. The presence of rGO imparted PI/HAP0.9/rGO with great photothermal conversion performance, which could further enhance the oil adsorption efficiency by reducing the oil viscosity with the in situ thermogenesis under sunlight, including crude oil with ultrahigh viscosity. In addition, the compressible PI/HAP/rGO presents excellent thermal stability and fire retardancy, allowing for reusability through convenient squeezing and combustion processes. Therefore, the prepared PI/HAP0.9/rGO is expected to be ideal adsorbent materials for clean-up and recovery of viscous oil spills exposed to sunlight.

## Materials and Methods

### Materials

*N*-*N*-dimethylacetamide (DMAC; 99%), triethylamine (TEA; 99%), sodium dihydrogen phosphate dihydrate (NaH_2_PO_4_·2H_2_O, >98%), sodium hydroxide (NaOH, FCC/NF), methanol (Laboratory), kerosene (reagent), hexane (reagent), toluene (reagent), dimethyl sulfoxide (DMSO), dichloromethane (residue analysis), and calcium chloride (CaCl_2_, >96%) were purchased from Fisher Scientific. 4,4′-Oxydianiline (ODA; 97%), pyromellitic dianhydride (PMDA; 97%), hydroxyapatite nanoparticles, and oleic acid (OA; technical grade) were purchased from Sigma-Aldrich. Graphite powders were purchased from Alfa Aesar Chemicals. Pump oil was purchased from Mastercool. Corn oil was purchased from Loblaws. Engine oil (0W-20) was purchased from Red Line. Engine oil (10W-30) was purchased from Valvoline. All chemicals were used as received without further purification.

### Preparation of PAA

The synthesis of PAA was a simple polycondensation method [43]. Briefly, in a nitrogen atmosphere, 4.31 g of ODA was added into 51 ml of DMAC in a 3-necked flask, which was immersed in an ice/water bath. The mixture was mechanically stirred for 20 min to fully dissolve the ODA. After that, 4.69 g of PMDA was added to the solution in batches. After the mixture was stirred for 3 h, 3 ml of TEA was injected into the flask and the stirring was kept for 4 h. After the reaction was completed, the viscous solution was poured into deionized water cooled with ice. The gel-like product was washed 4 times and frozen for 12 h at −18 °C. Finally, PAA was obtained after freeze drying for 48 h in a freeze dryer.

### Preparation of hydroxyapatite nanowires

The synthesis method of HAPnws refers to the reports of Zhu’s groups [36,37]. Typically, 62.4 g of OA, 31.67 g of methanol, and 90 ml of deionized (DI) water were added into a 500-ml beaker and uniformly mixed by magnetic stirring at 50 °C. Then, NaOH solution (100 ml of water with 7 g of NaOH), CaCl_2_ solution (100 ml of water with 2.22 g of CaCl_2_), and NaH_2_PO_4_·2H_2_O solution (100 ml of water with 6.27 g of NaH_2_PO_4_·2H_2_O) were added to the beaker sequentially every 20 min. Then, the hydrothermal reaction was conducted by adding the above mixture into a 1-l Teflon-lined hydrothermal autoclave and heated to 180 °C for 24 h in the oven. When the temperature cooled to room temperature, the slurry in the autoclave was washed with 500 ml of ethanol, and the white solid was obtained by centrifugation. To fully remove the unreacted ions and OA, the obtained white solid was washed with DI water 3 times, washed with a mixture of ethanol and NaOH solution 3 times, and finally washed with a substantial amount of water to reach a neutral pH of the filtrate. The washed white solid was dried at 60 °C before further use.

### Preparation of the PI/HAP/rGO aerogel

GO was synthesized via the modified Hummer’s method [45]. To fabricate the PI/HAP/rGO, 5 ml of GO solution with a concentration of 3 mg/ml was prepared via sonication in an ice bath for 1 h first. Then, 0.104 g of TEA and 0.15 g of PAA were added into the GO dispersion and magnetically stirred for 10 min to form a uniform solution. Different amounts of HAPnws (0, 0.015, 0.03, 0.045, 0.06, and 0.075 g) were added to the mixture and further magnetically stirred for 30 min. After that, the mixture solution was poured into a homemade directional freezing device to freeze the solution. The frozen sample was transferred to the freeze dryer and dried for 48 h at −56 °C. The obtained aerogel (PAA/HAP/GO) was placed in a tube furnace for the thermal reduction and imidization process. The temperature of the tube furnace was increased from the room temperature at the rate of 10 °C/min and maintained at 100, 200, and 300 °C for 1 h, respectively. After cooling to room temperature, the PI/HAP*x*/rGO (*x* = 0.3, 0.6, 0.9, 1.2, and 1.5, indicating the concentration of HAPnws) was obtained. The mixture solution of PAA, HAPnws, and GO was frozen in a refrigerator at −18 °C overnight, freeze dried, and thermal treated to prepare PI/HAP/rGO with a UP structure. For comparison, HAPnws were replaced by hydroxyapatite nanoparticles to prepare the composite aerogel, denoted as PI/HAP-NP/rGO.

### Characterization of the PI/HAP/rGO aerogel

SEM images and energy-dispersive x-ray spectroscopy element (EDX) mapping results were taken by Sigma EVO M10 (ZESSI, Germany). AFM imaging was obtained on ICON (Bruker, Germany) via the mapping mode. FTIR spectra were recorded by Nicolet iS50 (Thermo Fisher Scientific, USA). Raman spectroscopy spectra were recorded by a Renishaw InVia Raman microscope (Renishaw, UK). XPS spectra were obtained with Kratos AXIS Ultra (Kratos, UK). The rheological properties were tested by a rheometer (TA Instruments, AR-G2). The mechanical properties were tested by an AGS-X universal tensile testing machine (Shimadzu, Japan). The water contact angle was measured by a contact angle goniometer (ramé-hart instrument, NJ).

### Oil adsorption test

To test the oil adsorption speed, the aerogel samples were prepared with a size of ~12.5 mm in diameter and ~3 cm in length. Organic liquid (5 ml) was added to a rectangular container with a size of 3.5 cm × 3.5 cm. Then, the aerogel samples were placed in the center of the container. The adsorption process was recorded by a digital camera. The frame at different times of the video was taken and analyzed with ImageJ software to record the variation of liquid height with time.

### Flame retardancy test

Different aerogel samples were placed above the blowtorch and burned for 1 min to test their flame retardancy. The compressive test of the burned aerogel samples was conducted by an AGS-X universal tensile testing machine (Shimadzu, Japan). Adsorption–combustion test was performed using hexane as the flammable liquid. TGA was conducted with TGA Q500 (TA, USA).

### Photothermal property test

The temperature and the infrared photos were recorded with a FLIR-E6390 infrared camera (Teledyne FLIR, USA). Sunlight was simulated via a PLS-SXE300 xenon lamp source sun simulator (PerfectLight, China). The crude oil used in the solar-assisted adsorption test was obtained from JACOS (Athabasca area), with a density of 950 kg m^−3^.

## Data Availability

Data supporting the findings of this study can be obtained from the corresponding author upon request.

## Supplementary Materials

Supplementary 1 Figs. S1 to S10 Table S1 Movie S1 References

## References

[1] Soares MD, Teixeira CEP, Bezerra LEA, Paiva SV, Tavares TCL, Garcia TM, de Araújo JT, Campos CC, Ferreira SMC, Matthews-Cascon H, et al. Oil spill in South Atlantic (Brazil): Environmental and governmental disaster. Mar Policy. 2020;115: Article 103879.

[2] Yu HY, Wu M, Duan G, Gong X. One-step fabrication of eco-friendly superhydrophobic fabrics for high-efficiency oil/water separation and oil spill cleanup. Nanoscale. 2022;14(4):1296–1309. 35006232 10.1039/d1nr07111d

[3] Wong KFV, Barin E. Oil spill containment by a flexible boom system. Spill Sci Tech Bull. 2003;8(5–6):509–520.

[4] Bullock RJ, Perkins RA, Aggarwal S. In-situ burning with chemical herders for Arctic oil spill response: Meta-analysis and review. Sci Total Environ. 2019;675:705–716. 31042623 10.1016/j.scitotenv.2019.04.127

[5] Varjani SJ. Microbial degradation of petroleum hydrocarbons. Bioresour Technol. 2017;223:277–286. 27789112 10.1016/j.biortech.2016.10.037

[6] Qi BH, Hu X, Cui S, Liu H, Li Y, Li Y, Lu J, Bao M. Rapid fabrication of superhydrophobic magnetic melt-blown fiber felt for oil spill recovery and efficient oil-water separation. Sep Purif Technol. 2023;306: Article 122486.

[7] Nikkhah AA, Zilouei H, Asadinezhad A, Keshavarz A. Removal of oil from water using polyurethane foam modified with nanoclay. Chem Eng J. 2015;262:278–285.

[8] Jiang JX, Zhang Q, Zhan X, Chen F. A multifunctional gelatin-based aerogel with superior pollutants adsorption, oil/water separation and photocatalytic properties. Chem Eng J. 2019;358:1539–1551.

[9] Luo Z, Wang X, Yang D, Zhang S, Zhao T, Qin L, Yu ZZ. Photothermal hierarchical carbon nanotube/reduced graphene oxide microspherical aerogels with radially orientated microchannels for efficient cleanup of crude oil spills. J Colloid Interface Sci. 2020;570:61–71. 32142904 10.1016/j.jcis.2020.02.097

[10] Chao WX, Wang S, Li Y, Cao G, Zhao Y, Sun X, Wang C, Ho SH. Natural sponge-like wood-derived aerogel for solar-assisted adsorption and recovery of high-viscous crude oil. Chem Eng J. 2020;400: Article 125865.

[11] Bayat A, Aghamiri SF, Moheb A, Vakili-Nezhaad GR. Oil spill cleanup from sea water by sorbent materials. Chem Eng Technol. 2005;28(12):1525–1528.

[12] Ammann J, Ruch P, Michel B, Studart AR. High-power adsorption heat pumps using magnetically aligned zeolite structures. ACS Appl Mater Interfaces. 2019;11(27):24037–24046. 31251575 10.1021/acsami.9b04692

[13] Sai HZ, Xing L, Xiang J, Cui L, Jiao J, Zhao C, Li Z, Li F. Flexible aerogels based on an interpenetrating network of bacterial cellulose and silica by a non-supercritical drying process. J Mater Chem A. 2013;1:7963–7970.

[14] Liu H, Zhai W, Park CB. Biomimetic hydrophobic plastic foams with aligned channels for rapid oil absorption. J Hazard Mater. 2022;437: Article 129346. 35716573 10.1016/j.jhazmat.2022.129346

[15] Wu MB, Huang S, Liu TY, Wu J, Agarwal S, Greiner A, Xu ZK. Compressible carbon sponges from delignified wood for fast cleanup and enhanced recovery of crude oil spills by joule heat and photothermal effect. Adv Funct Mater. 2021;31(3):2006806.

[16] Shao G, Hanaor DAH, Shen X, Gurlo A. Freeze casting: From low-dimensional building blocks to aligned porous structures—A review of novel materials, methods, and applications. Adv Mater. 2020;32(17): Article e1907176. 32163660 10.1002/adma.201907176

[17] Shahbazi M-A, Ghalkhani M, Maleki H. Directional freeze-casting: A bioinspired method to assemble multifunctional aligned porous structures for advanced applications. Adv Eng Mater. 2020;22(7):2000033.

[18] Wang L, Song P, Lin CT, Kong J, Gu J. 3D shapeable, superior electrically conductive cellulose nanofibers/Ti_3_C_2_T_x_ MXene aerogels/epoxy nanocomposites for promising EMI shielding. Research. 2020;2020:4093732. 32613198 10.34133/2020/4093732 PMC7317662

[19] Ma XZ, Zhang C, Gnanasekar P, Xiao P, Luo Q, Li S, Qin D, Chen T, Chen J, Zhu J, et al. Mechanically robust, solar-driven, and degradable lignin-based polyurethane adsorbent for efficient crude oil spill remediation. Chem Eng J. 2021;415: Article 128956.

[20] Zhang C, Wu MB, Wu BH, Yang J, Xu ZK. Solar-driven self-heating sponges for highly efficient crude oil spill remediation. J Mater Chem A. 2018;6:8880–8885.

[21] Wu M, Shi Y, Chang J, Li R, Ong C, Wang P. Sunlight induced rapid oil absorption and passive room-temperature release: An effective solution toward heavy oil spill cleanup. Adv Mater Interfaces. 2018;5(14):1800412.

[22] Chang J, Shi Y, Wu M, Li R, Shi L, Jin Y, Qing W, Tang C, Wang P. Solar-assisted fast cleanup of heavy oil spills using a photothermal sponge. J Mater Chem A. 2018;6:9192–9199.

[23] Ma X, Chen K, Li S, Gnanasekar P, Zhong Y, An Y, Luo Q, Huang Q, Zhu J, Chen J, et al. Degradable Ti_3_C_2_T_x_ MXene nanosheets containing a lignin polyurethane photothermal foam (LPUF) for rapid crude oil cleanup. ACS Appl Nano Mater. 2022;5(2):2848–2858.

[24] Xue TT, Yang F, Zhao X, He F, Wang Z, Wali Q, Fan W, Liu T. Portable solar interfacial evaporator based on polyimide nanofiber aerogel for efficient desalination. Chem Eng J. 2023;461: Article 141909.

[25] Yang WK, Liu H, du H, Zhang M, Wang C, Yin R, Pan C, Liu C, Shen C. Robust and superelastic spider web-like polyimide fiber-based conductive composite aerogel for extreme temperature-tolerant linear pressure sensor. Sci China Mater. 2023;66:2829–2842.

[26] Yao KQ, Song C, Fang H, Wang F, Chen L, Jiang S, Zha G, Hou H. Freezing-extraction/vacuum-drying method for robust and fatigue-resistant polyimide fibrous aerogels and their composites with enhanced fire retardancy. Engineering. 2023;21:152–161.

[27] Wang YM, Zeng X, Wang W, Zhou P, Zhang R, Chen H, Liu G. Superhydrophobic polyimide/cattail-derived active carbon composite aerogels for effective oil/water separation. Sep Purif Technol. 2023;308: Article 122994.

[28] Ruan K, Guo Y, Lu C, Shi X, Ma T, Zhang Y, Kong J, Gu J. Significant reduction of interfacial thermal resistance and phonon scattering in graphene/polyimide thermally conductive composite films for thermal management. Research. 2021;2021:8438614. 33718876 10.34133/2021/8438614 PMC7931127

[29] Parale VG, Kim T, Choi H, Phadtare VD, Dhavale RP, Kanamori K, Park HH. Mechanically strengthened aerogels through multiscale, multicompositional, and multidimensional approaches: A review. Adv Mater. 2024;36(18): Article e2307772. 37916304 10.1002/adma.202307772

[30] Qiao SY, Yan J, Wang Z, Wang Y, Yu J, Hu Z. Tough and lightweight polyimide/cellulose nanofiber aerogels with hierarchical porous structures as an efficient air purifier. Sep Purif Technol. 2023;325: Article 124668.

[31] Dai W, Yu J, Wang Y, Song Y, Alam FE, Nishimura K, Lin CT, Jiang N. Enhanced thermal conductivity for polyimide composites with a three-dimensional silicon carbide nanowire@graphene sheets filler. J Mater Chem A. 2015;3:4884–4891.

[32] Jiang CC, Chen J, Lai X, Li H, Zeng X, Zhao Y, Zeng Q, Gao J, Wu Z, Qiu Y. Mechanically robust and multifunctional polyimide/MXene composite aerogel for smart fire protection. Chem Eng J. 2022;434: Article 134630.

[33] Xu LL, Chen H, Zheng P, Zheng H, Chen Z, Liu Z, Li P, Zhao X, Peng Q, He X. Lightweight and mechanically robust MXene/polyimide/silver nanowire composite aerogels for flexible piezoresistive sensors. ACS Appl Nano Mater. 2023;6(20):19098–19107.

[34] Zheng ZH, Liu H, Wu D, Wang X. Polyimide/MXene hybrid aerogel-based phase-change composites for solar-driven seawater desalination. Chem Eng J. 2022;440: Article 135862.

[35] Zhang YG, Zhu YJ, Xiong ZC, Wu J, Chen F. Bioinspired ultralight inorganic aerogel for highly efficient air filtration and oil-water separation. ACS Appl Mater Interfaces. 2018;10(15):13019–13027. 29611706 10.1021/acsami.8b02081

[36] Xiong ZC, Zhu YJ, Wang ZY, Chen YQ, Yu HP. Tree-inspired ultralong hydroxyapatite nanowires-based multifunctional aerogel with vertically aligned channels for continuous flow catalysis, water disinfection, and solar energy-driven water purification. Adv Funct Mater. 2021;32(9):2106978.

[37] Huang P, Fang W, Yang L, Sun Y, Yang H, Chen XZ, Zeng H. Ultralong hydroxyapatite nanowires-based flow-through reactor with high loading of silver nanoparticles for fast continuous catalytic reduction of organic dyes and disinfection of wastewater. Chem Eng J. 2023;475: Article 146305.

[38] Xiong L, Zheng W, Cao S, Zheng Y. Organic-inorganic double-gel system thermally insulating and hydrophobic polyimide aerogel. Polymers. 2022;14(14):2818. 35890593 10.3390/polym14142818 PMC9321330

[39] Hou X, Zhang R, Fang D. Flexible MCNTs cross-linked polyimide membranes with high light absorbance and hierarchical pore distribution for photo-thermal conversion in solar water evaporation. Carbon. 2022;187:310–320.

[40] Yang J, Li HN, Zhang X, Zhu CY, Yu HH, Xu ZK. Janus membranes for fast-mass-transfer separation of viscous ionic liquids from emulsions. J Membr Sci. 2021;637: Article 119643.

[41] Pandey RP, Thakur AK, Shahi VK. Sulfonated polyimide/acid-functionalized graphene oxide composite polymer electrolyte membranes with improved proton conductivity and water-retention properties. ACS Appl Mater Interfaces. 2014;6(19):16993–17002. 25207457 10.1021/am504597a

[42] Wu P, Zhang B, Yu Z, Zou H, Liu P. Anisotropic polyimide aerogels fabricated by directional freezing. J Appl Polym Sci. 2019;136(11):47179.

[43] Wang NN, Wang H, Wang YY, Wei YH, Si JY, Yuen ACY, Xie JS, Yu B, Zhu SE, Lu HD, et al. Robust, lightweight, hydrophobic, and fire-retarded polyimide/MXene aerogels for effective oil/water separation. ACS Appl Mater Interfaces. 2019;11(43):40512–40523. 31577120 10.1021/acsami.9b14265

[44] Washburn EW. The dynamics of capillary flow. Phys Rev. 1921;17:273–283.

[45] Jiříčková A, Jankovský O, Sofer Z, Sedmidubský D. Synthesis and applications of graphene oxide. Materials. 2022;15(3):920. 35160865 10.3390/ma15030920 PMC8839209

[46] Chen XY, Liu H, Zheng Y, Zhai Y, Liu X, Liu C, Mi L, Guo Z, Shen C. Highly compressible and robust polyimide/carbon nanotube composite aerogel for high-performance wearable pressure sensor. ACS Appl Mater Interfaces. 2019;11(45):42594–42606. 31618002 10.1021/acsami.9b14688

[47] Yan YX, Ge F, Qin Y, Ruan M, Guo Z, He C, Wang Z. Ultralight and robust aerogels based on nanochitin towards water-resistant thermal insulators. Carbohydr Polym. 2020;248: Article 116755. 32919557 10.1016/j.carbpol.2020.116755

[48] Guo HQ, Meador MAB, McCorkle L, Quade DJ, Guo J, Hamilton B, Cakmak M, Sprowl G. Polyimide aerogels cross-linked through amine functionalized polyoligomeric silsesquioxane. ACS Appl Mater Interfaces. 2011;3(2):546–552. 21294517 10.1021/am101123h

[49] Li HX, Zhao F, Peng T, Jiang C, Liu H, He Y, He D. Robust, lightweight gelatin composite aerogel with outstanding thermal insulation. J Mater Sci. 2022;57:14835–14847.

[50] Meador MAB, Wright S, Sandberg A, Nguyen BN, Van Keuls FW, Mueller CH, Rodríguez-Solís R, Miranda FA. Low dielectric polyimide aerogels as substrates for lightweight patch antennas. Abstr Pap Am Chem Soc. 2013;245(11):6346–6353. 10.1021/am301985s 23134844

[51] Zhang XH, Ni X, Li C, You B, Sun G. Co-gel strategy for preparing hierarchically porous silica/polyimide nanocomposite aerogel with thermal insulation and flame retardancy. J Mater Chem A. 2020;8:9701–9712.

