# Supplementary material for: Highly Robust, Compressible, Anisotropic, and Fire-Retardant Polyimide/Hydroxyapatite Nanowires/Reduced Graphene Oxide Aerogel for Rapid Adsorption of Viscous Oil Assisted by Sunlight
Source: Research (Wash D C). 2024 Oct 29;7:0512. doi: 10.34133/research.0512 (PMC11520237; doi:10.34133/research.0512)
Supplement: Supplementary 1 — Figs. S1 to S10 Table S1 Movie S1 References [file research.0512.f1.zip › Supporting information.docx]

**Supporting Information**

**Highly Robust, Compressible, Anisotropic, and Fire-retardant Polyimide/Hydroxyapatite nanowires/rGO Aerogel for Rapid Adsorption of Viscous Oil Assisted by Sunlight**

*Pan Huang^1^, Yongxiang Sun^1^, Lin Yang^1^, Haoyu Yang^1^, Ying Hu^2^, Jifang Liu^3^, Xuwen Peng^1,4,^*, Hongbo Zeng^1,^**

*^1^ Department of Chemical and Materials Engineering, University of Alberta, Edmonton, T6G 1H9, Alberta, Canada.*

*^2^Heavy Machinery Engineering Research Center of Education Ministry, Taiyuan University of Science and Technology, Taiyuan 030024, China*

*^3^Cancer Center, The Fifth Affiliated Hospital, Guangzhou Medical University, 510700 Guangzhou, People’s Republic of China.*

*^4^ Department of Chemical Engineering, Tsinghua University, Beijing 10084, China*

**Corresponding authors. Xuwen Peng;* [*xuwen@tsinghua.edu.cn*](mailto:xuwen@tsinghua.edu.cn) *(X.P.) and Hongbo Zeng;* [*hongbo.zeng@ualberta.ca*](mailto:hongbo.zeng@ualberta.ca) *(H.Z.)*


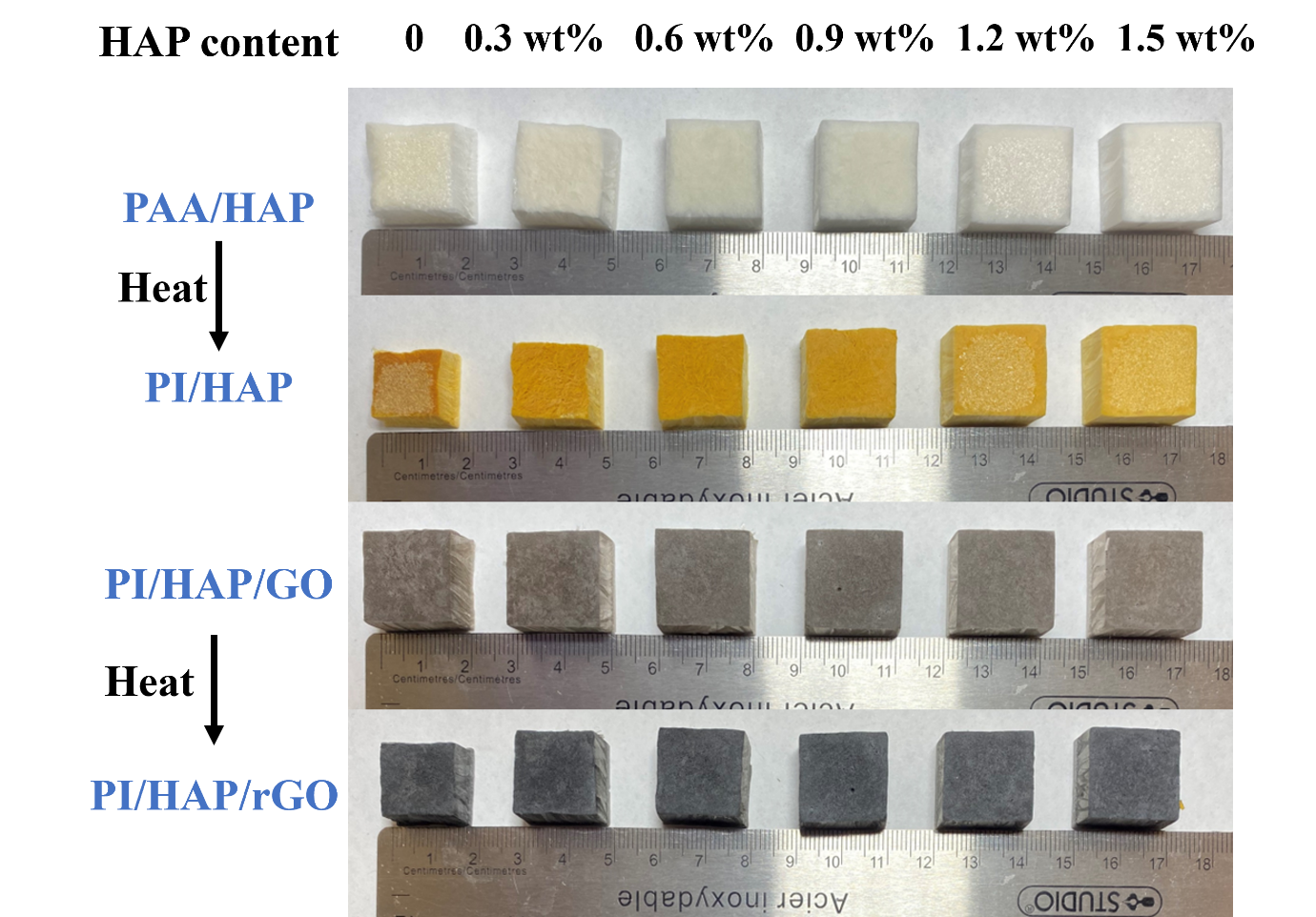


**Figure S1.** Photos of PI/HAP and PI/HAP/rGO with different contents of HAPnws before and after the thermal imidization process.


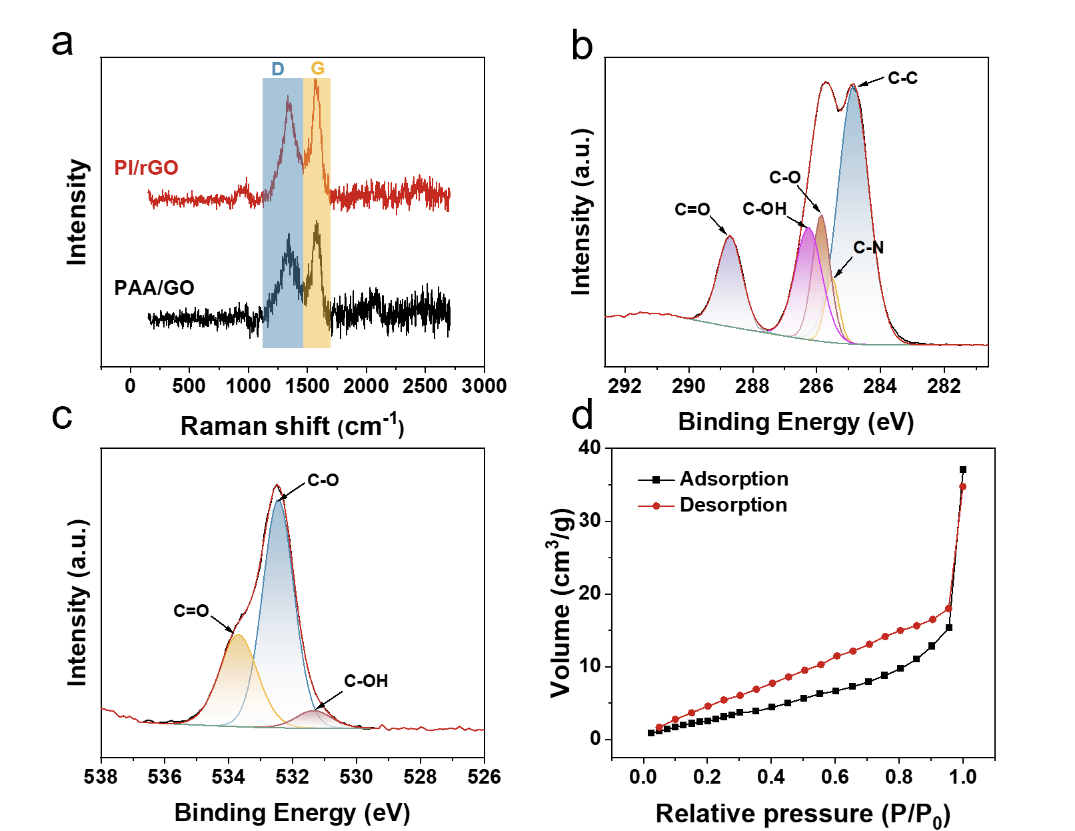


**Figure S2.** (a) Raman spectra of PAA/GO and PI/rGO, (b) High-resolution XPS spectra of C 1s and (c) O 1s of PI/HAP0.9/rGO, (d) N_2_ adsorption-desorption isotherms of the PI/HAP0.9/rGO aerogel.


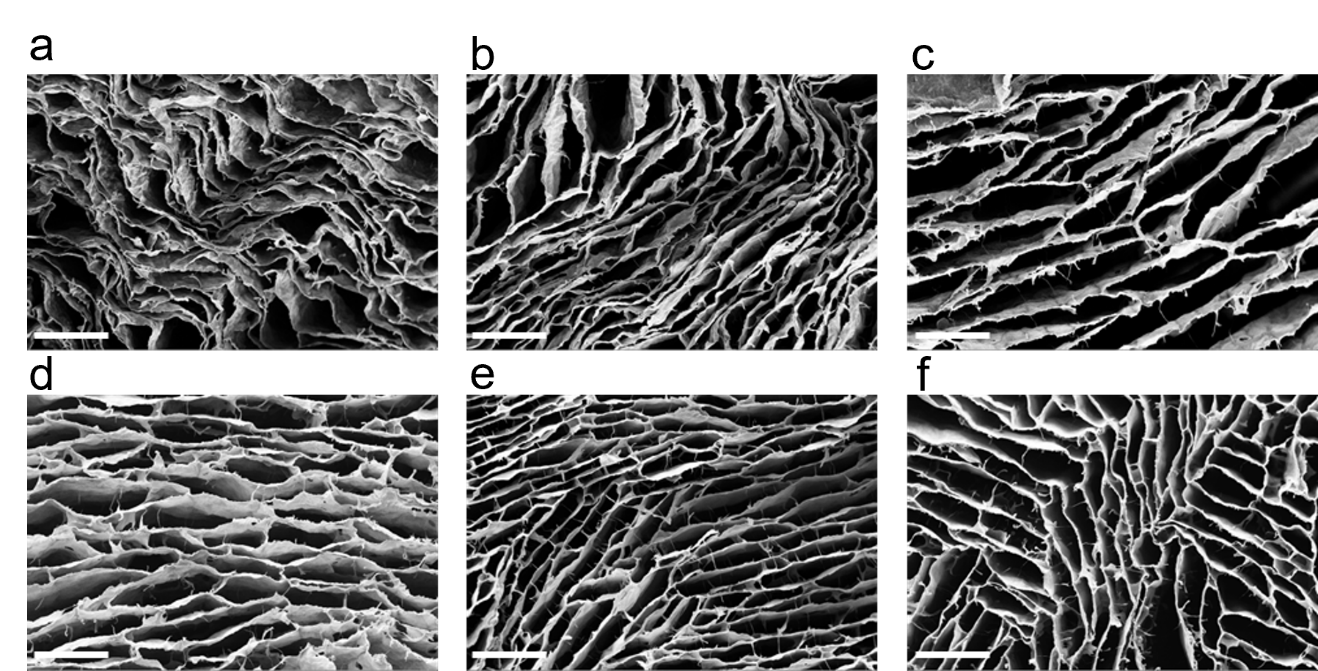


**Figure S3.** SEM images along the radial direction of PI/HAP/rGO with HAPnws content of (a) 0 wt%, (b) 0.3 wt%, (c) 0.6 wt%, (d) 0.9 wt%, (e) 1.2 wt%, (f) 1.5 wt%, the scale bar is 100 μm.

**
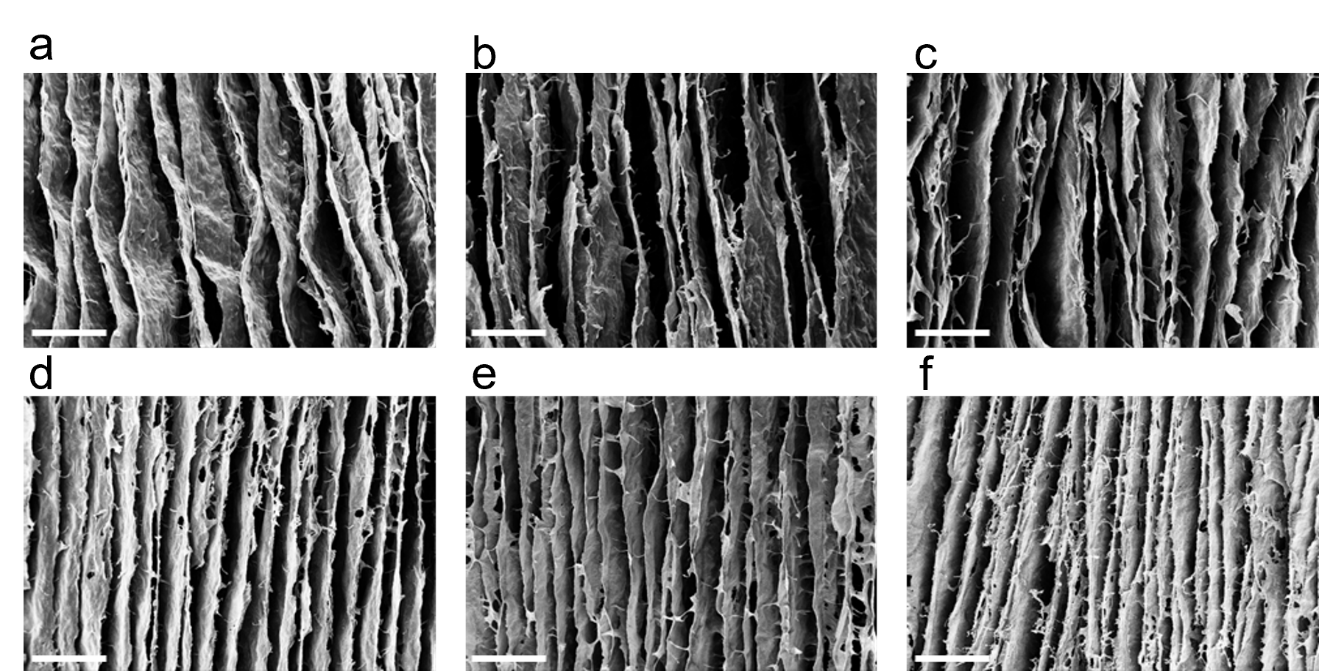
**

**Figure S4.** SEM images along the radial direction of PI/HAP/rGO with HAPnws content of (a) 0 wt%, (b) 0.3 wt%, (c) 0.6 wt%, (d) 0.9 wt%, (e) 1.2 wt%, (f) 1.5 wt%, the scale bar is 100 μm.


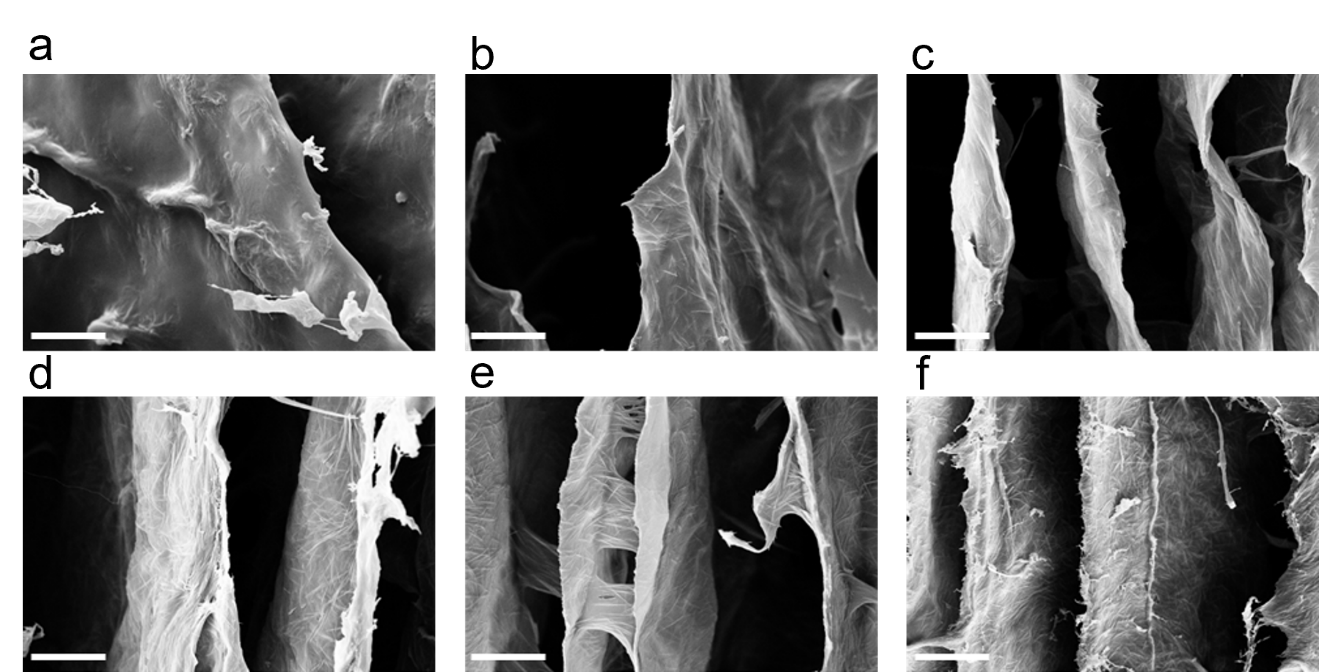


**Figure S5.** SEM images along the axial direction of PI/HAP/rGO with HAPnws content of (a) 0 wt%, (b) 0.3 wt%, (c) 0.6 wt%, (d) 0.9 wt%, (e) 1.2 wt%, (f) 1.5 wt%, the scale bar is 10 μm.


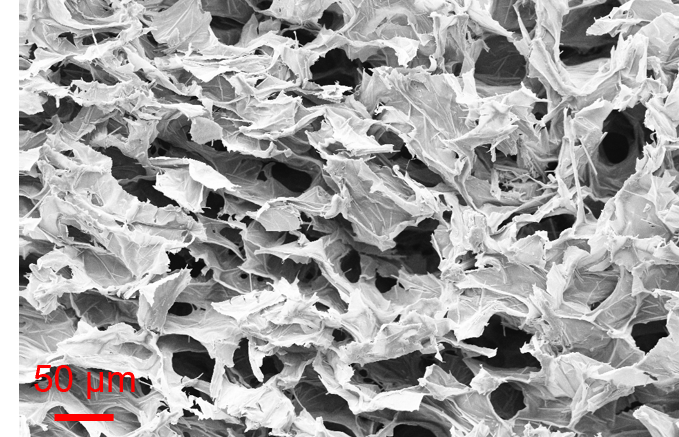


**Figure S6.** SEM image of PI/HAP0.9/rGO aerogel with uniform pore structure.


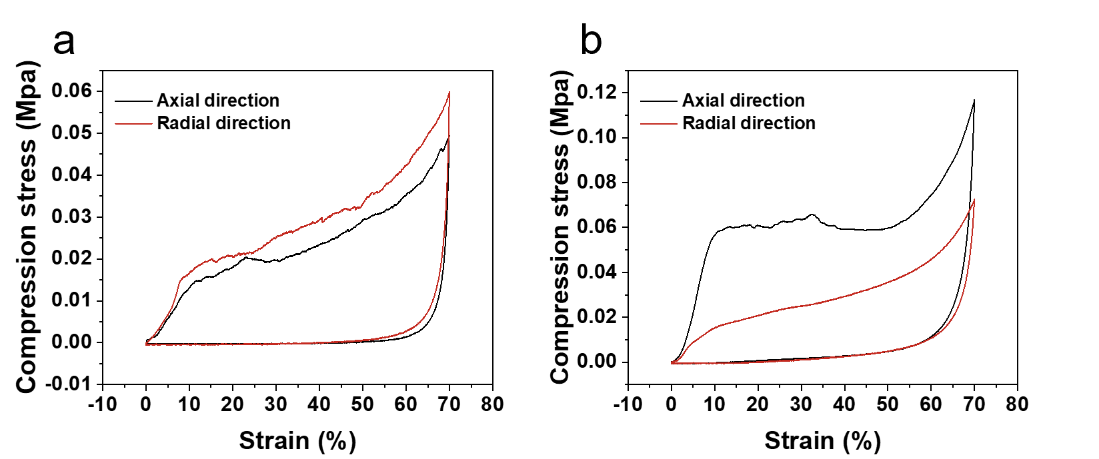


**Figure S7.** Compressive stress-strain curve of PI/HAP0.9/rGO with uniform pore structure (a) and PI/HAP-NP/rGO with directional channel structure.


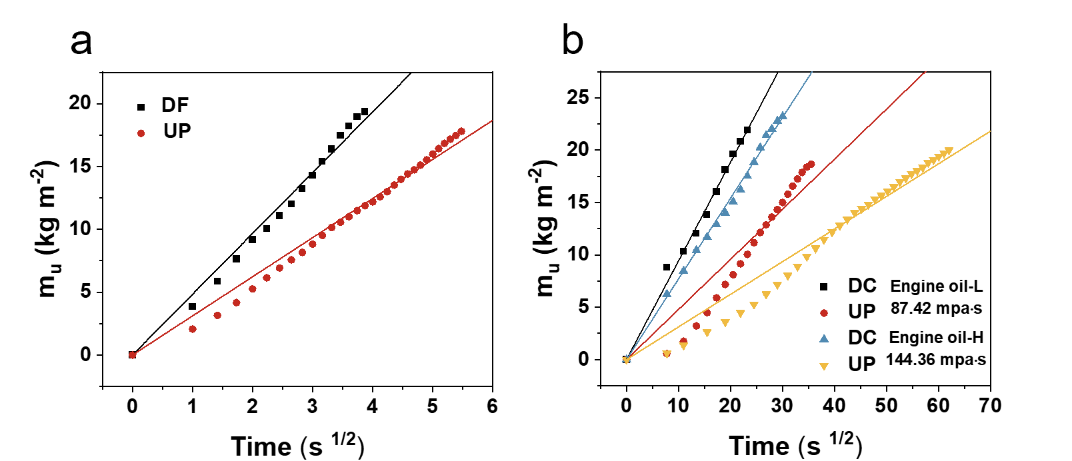


**Figure S8.** (a) $m_{u}$- t^½^ curves of PI/HAP0.9/rGO with DC and UP structures for the adsorption of Kerosene; (b) $m_{u}$- t^½^ curves of PI/HAP0.9/rGO with DC and UP structures for the adsorption of engine oil-L and engine oil-H.


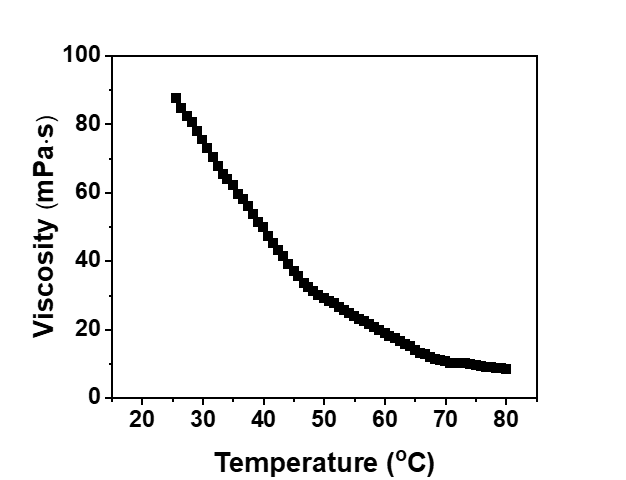


**Figure S9.** The viscosity of engine oil-L changed with temperature.

| Table S1. Adsorption coefficient Ks comparison of reported adsorbents in the literature | | | |
| --- | --- | --- | --- |
| Materials | Liquid viscosity | Ks  *Kg m^-2^ s^-1/2^* | Ref |
| PU sponge | Liquid paraffin, 11 mPa·s at 50 ℃ | 3.4 | ^[1]^ |
| PLA foams with aligned channel | Engine oil, 43.6 mPa·s at 25 ℃ | 1.56 | ^[2]^ |
| PLA foams with aligned channel | Silicone oil, 970 mPa·s at 25 ℃ | 0.31 |  |
| Graphene-wrapped sponge | Crude oil, 4500 mPa·s at 20 ℃ | 0.041 | ^[3]^ |
| HC-wood | Crude oil, 210 mPa·s at 85 ℃ | 0.27 | ^[4]^ |
| PI/HAP0.9/rGO | Engine oil, 87.42 mPa·s at 25 ℃ | 1.23 | This work |
|  | Crude oil, 11000 mPa·s at 25 ℃ | 0.25 |  |


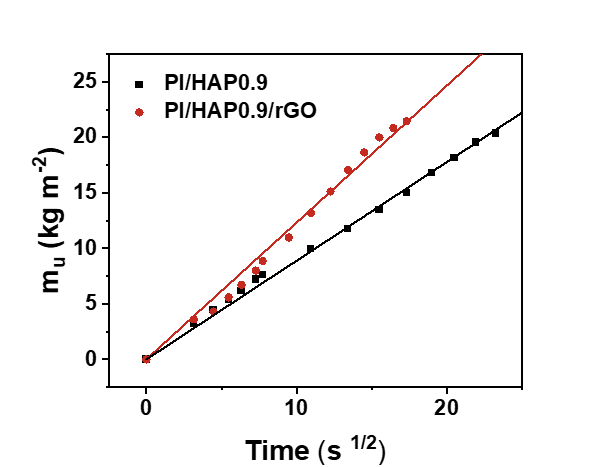


**Figure S10.** $m_{u}$- t^½^ curves of PI/HAP0.9 and PI/HAP0.9/rGO for the adsorption of engine oil-L with the assistance of sunlight.

**Video S1.** The burning process of PI and PI/HAP0.9/rGO adsorbed with hexane.

**References**

1 Cui Y, Wang YJ, Shao ZY, et al. Smart Sponge for Fast Liquid Absorption and Thermal Responsive Self-Squeezing. Advanced Materials 2020;32.

2 Liu H, Zhai W, Park CB. Biomimetic hydrophobic plastic foams with aligned channels for rapid oil absorption. J Hazard Mater 2022;437:129346.

3 Ge J, Shi LA, Wang YC, et al. Joule-heated graphene-wrapped sponge enables fast clean-up of viscous crude-oil spill. Nature Nanotechnology 2017;12:434-440.

4 Kuang YD, Chen CJ, Chen G, et al. Bioinspired Solar-Heated Carbon Absorbent for Efficient Cleanup of Highly Viscous Crude Oil. Advanced Functional Materials 2019;29.
