# Supplementary figures and images for: Highly Robust, Compressible, Anisotropic, and Fire-Retardant Polyimide/Hydroxyapatite Nanowires/Reduced Graphene Oxide Aerogel for Rapid Adsorption of Viscous Oil Assisted by Sunlight
Source: Research (Wash D C). 2024 Oct 29;7:0512. doi: 10.34133/research.0512 (PMC11520237; doi:10.34133/research.0512)

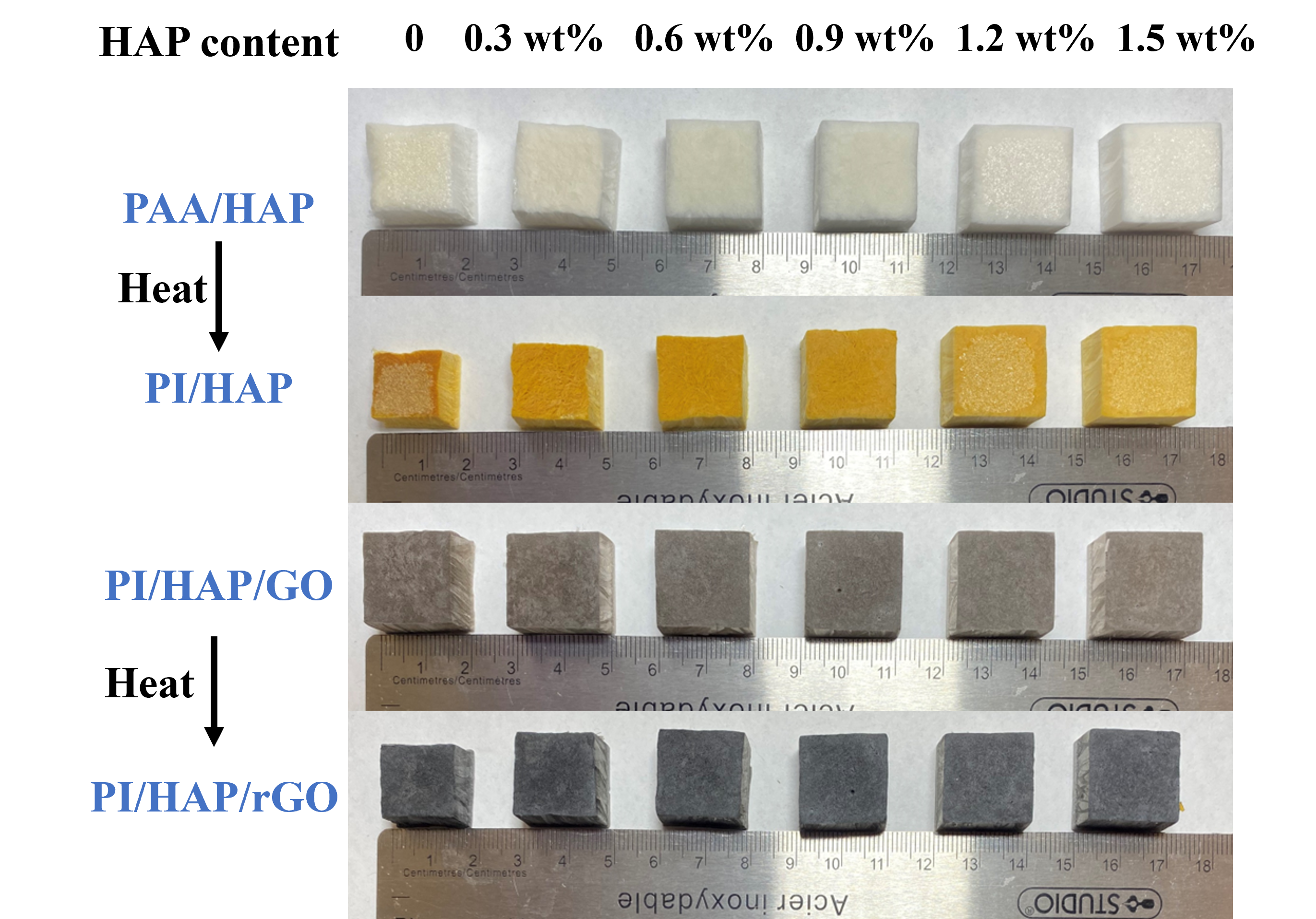

Supplement: Supplementary 1 — Figs. S1 to S10 Table S1 Movie S1 References [file research.0512.f1.zip › Figure S1.tif]

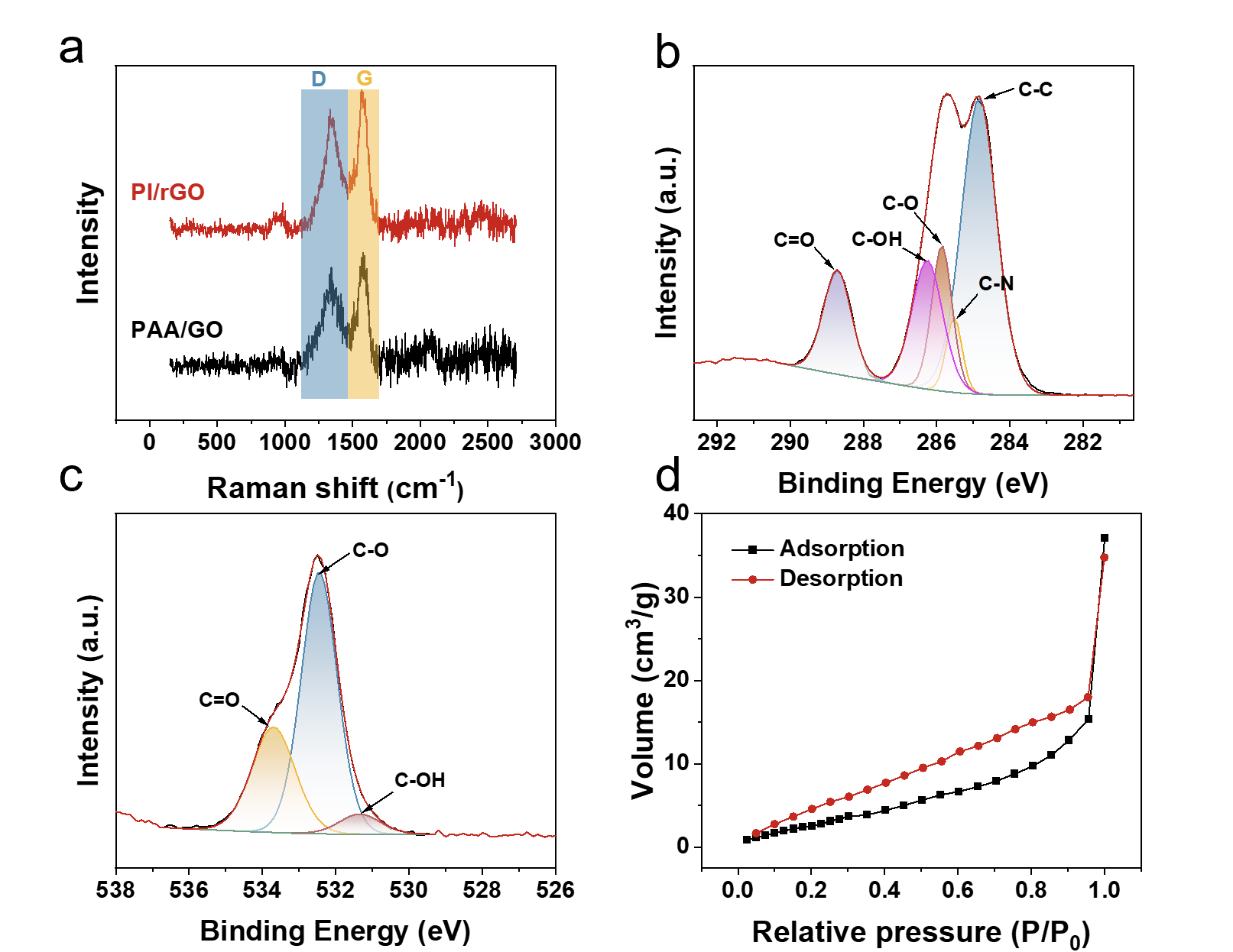

Supplement: Supplementary 1 — Figs. S1 to S10 Table S1 Movie S1 References [file research.0512.f1.zip › Figure S2.tif]

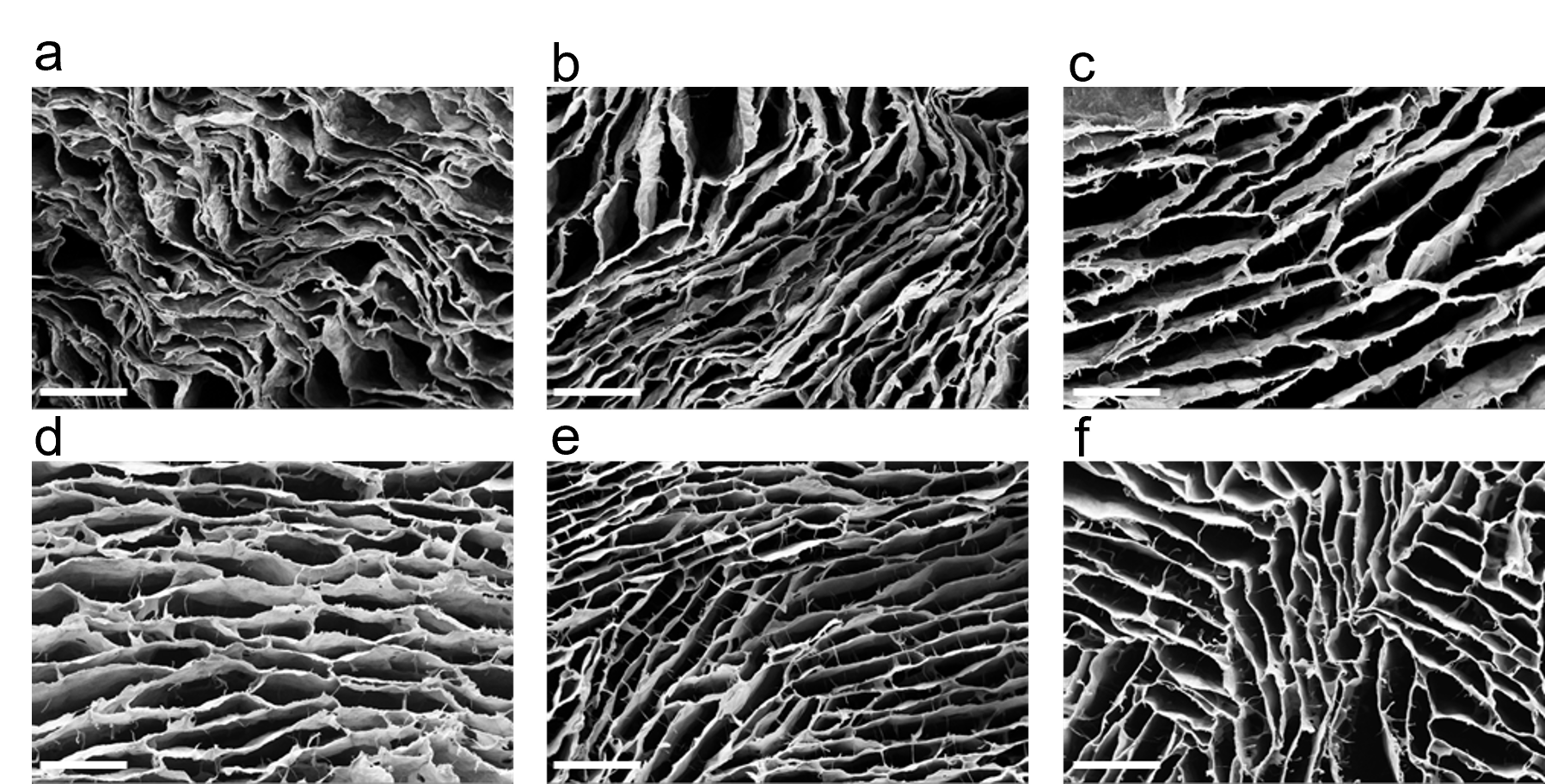

Supplement: Supplementary 1 — Figs. S1 to S10 Table S1 Movie S1 References [file research.0512.f1.zip › Figure S3.tif]

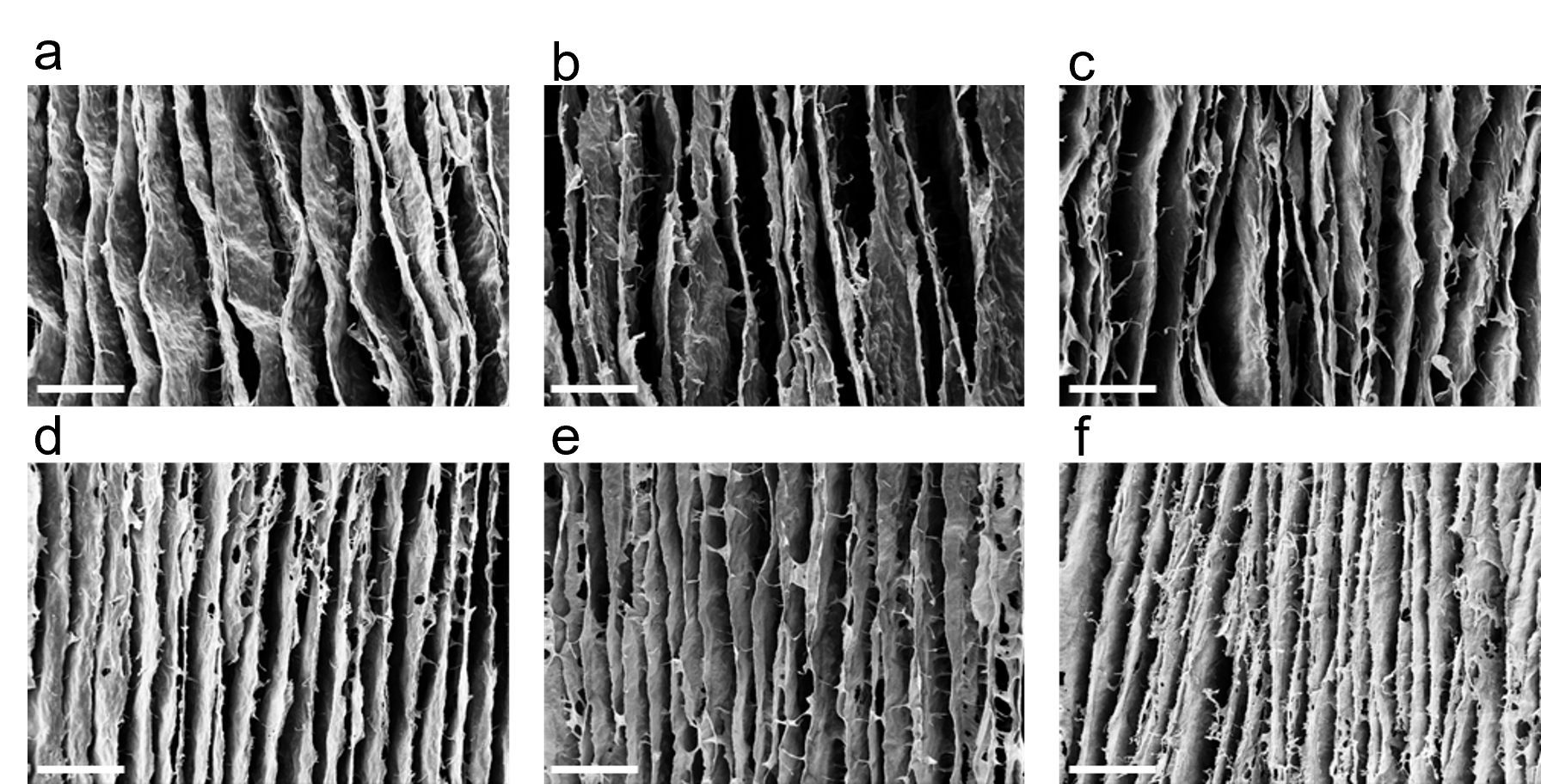

Supplement: Supplementary 1 — Figs. S1 to S10 Table S1 Movie S1 References [file research.0512.f1.zip › Figure S4.tif]

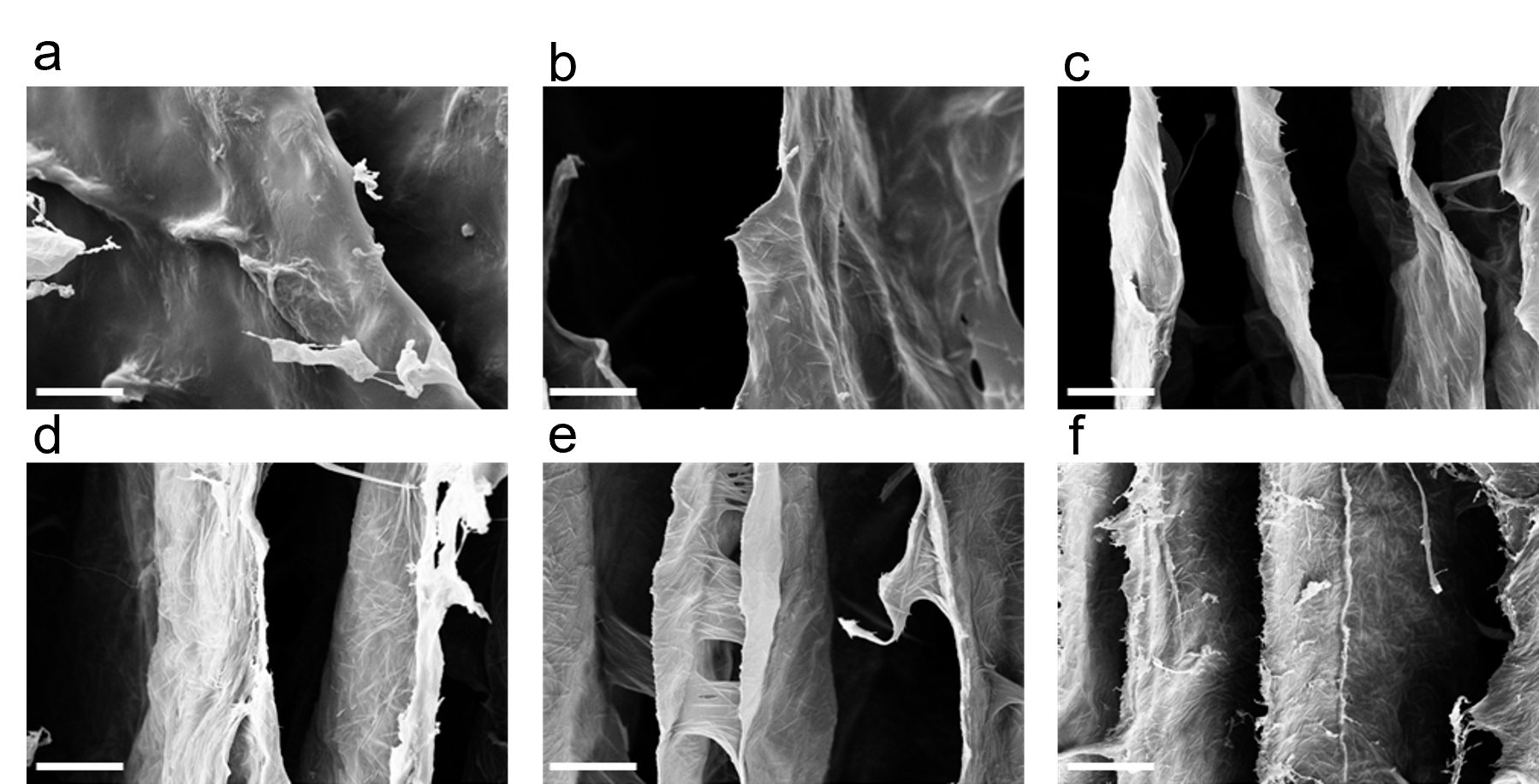

Supplement: Supplementary 1 — Figs. S1 to S10 Table S1 Movie S1 References [file research.0512.f1.zip › Figure S5.tif]

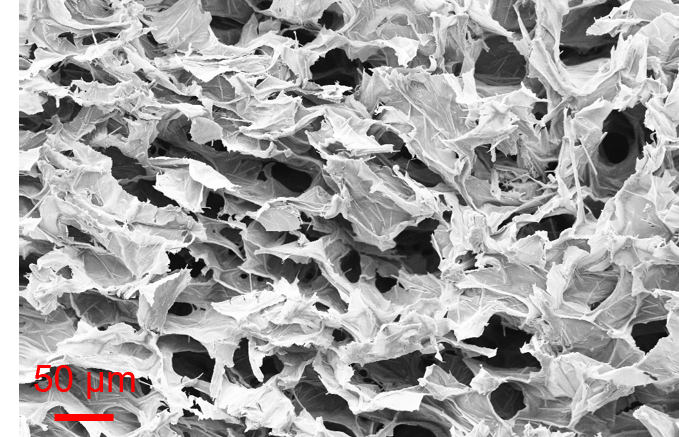

Supplement: Supplementary 1 — Figs. S1 to S10 Table S1 Movie S1 References [file research.0512.f1.zip › Figure S6.tif]

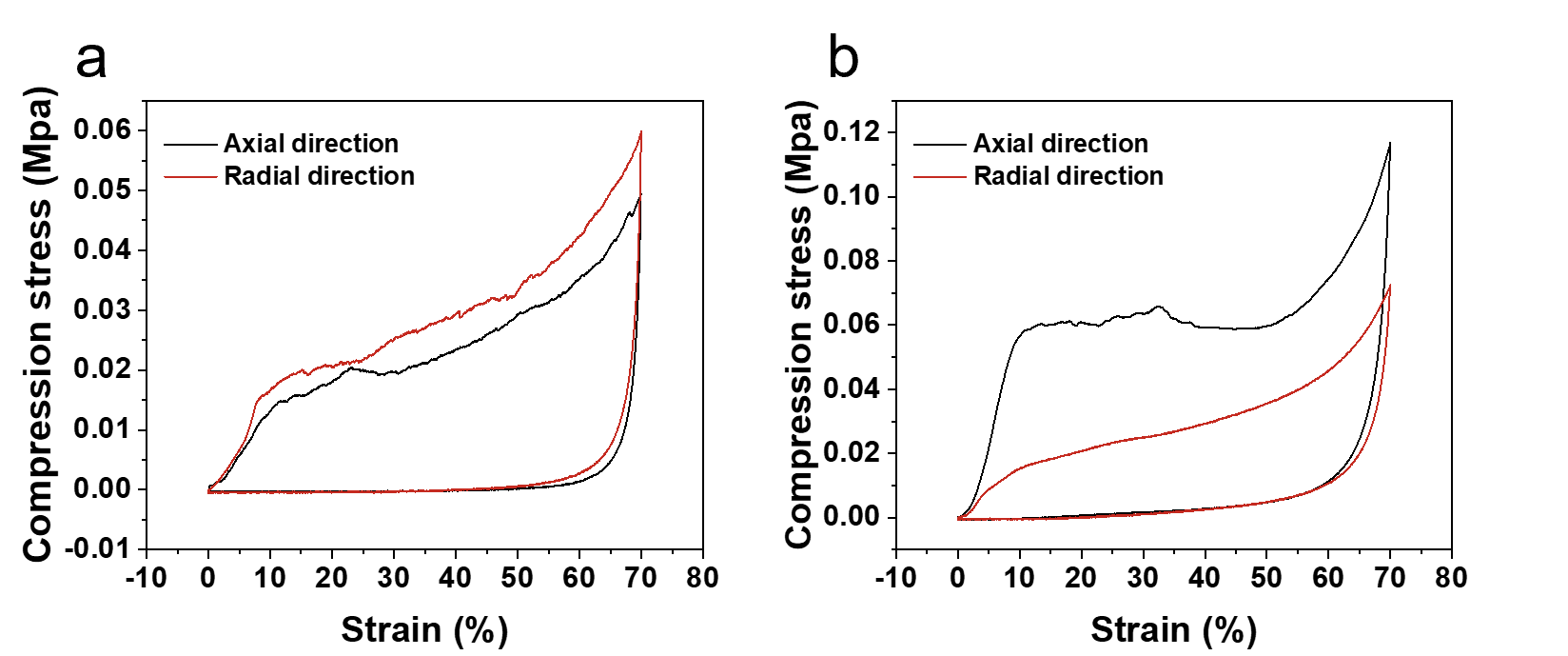

Supplement: Supplementary 1 — Figs. S1 to S10 Table S1 Movie S1 References [file research.0512.f1.zip › Figure S7.tif]

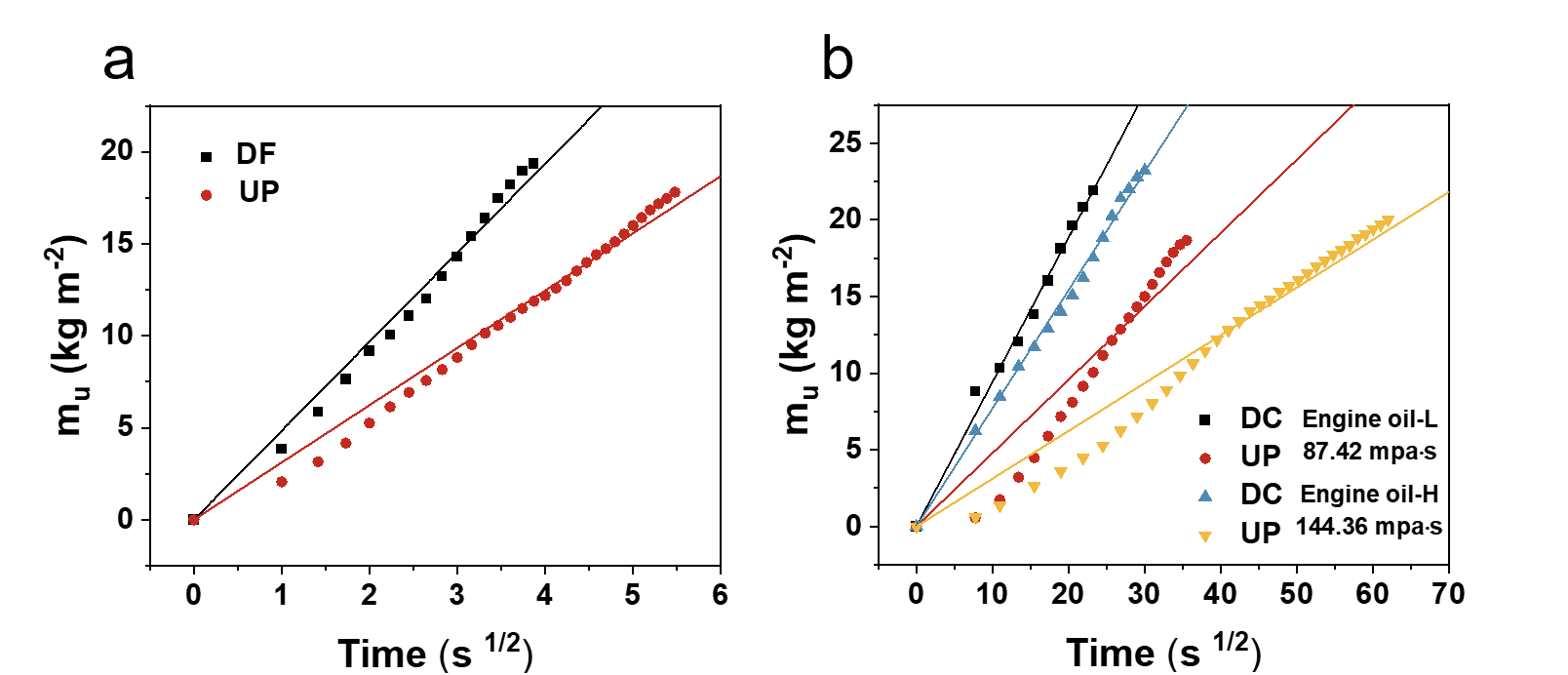

Supplement: Supplementary 1 — Figs. S1 to S10 Table S1 Movie S1 References [file research.0512.f1.zip › Figure S8.tif]

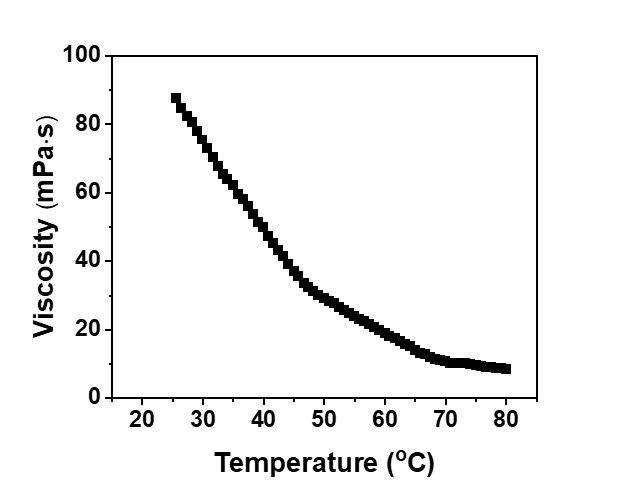

Supplement: Supplementary 1 — Figs. S1 to S10 Table S1 Movie S1 References [file research.0512.f1.zip › Figure S9.tif]

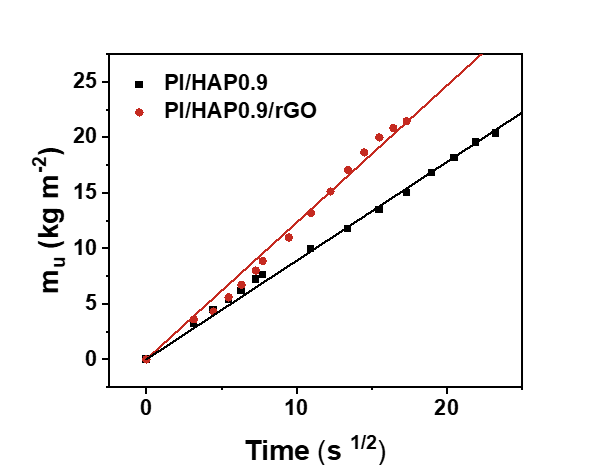

Supplement: Supplementary 1 — Figs. S1 to S10 Table S1 Movie S1 References [file research.0512.f1.zip › Figure S10.tif]
